# *De novo* synthesis of D- and L-fucosamine containing disaccharides

**DOI:** 10.3762/bjoc.9.38

**Published:** 2013-02-14

**Authors:** Daniele Leonori, Peter H Seeberger

**Affiliations:** 1 Department of Biomolecular Systems, Max Planck Institute of Colloids and Interfaces, Am Mühlenberg 1, 14476 Potsdam, Germany; 2 Institute for Chemistry and Biochemistry, Freie Universität Berlin, Arnimallee 22, 14195 Berlin, Germany

**Keywords:** *de novo* synthesis, fucosamine, glycan, *pseudomonas aeruginosa*, vaccine

## Abstract

The availability of rare monosaccharides that cannot be isolated from natural sources is currently limiting the access to the synthesis and the biological evaluation of complex bacterial cell-surface glycans. Here, we report the synthesis of D- and L-fucosamine building blocks by a *de novo* approach from L- and D-Garner aldehydes. These differentially protected monosaccharide building blocks were utilized to prepare disaccharides present on the surface of *Pseudomonas aeruginosa* bacteria.

## Introduction

Protein functions are directly influenced by their glycosylation patterns [1–2]. Therefore, an understanding of protein glycosylation is of utmost importance in order to develop new therapeutics [3–6]. The ability of bacteria to colonize human hosts and cause diseases is directly influenced by their capacity to synthesize glycoproteins and express them on cell surfaces. This evidence makes it particularly relevant especially for the identification of novel antibacterial agents as well as vaccines [7–9]. Those bacterial glycans often contain unusual monosaccharides that are not present in the human body. An immune response against these cell-surface glycans is the basis for the development of new vaccine candidates against bacterial infections [10–13].

**Figure 1:**
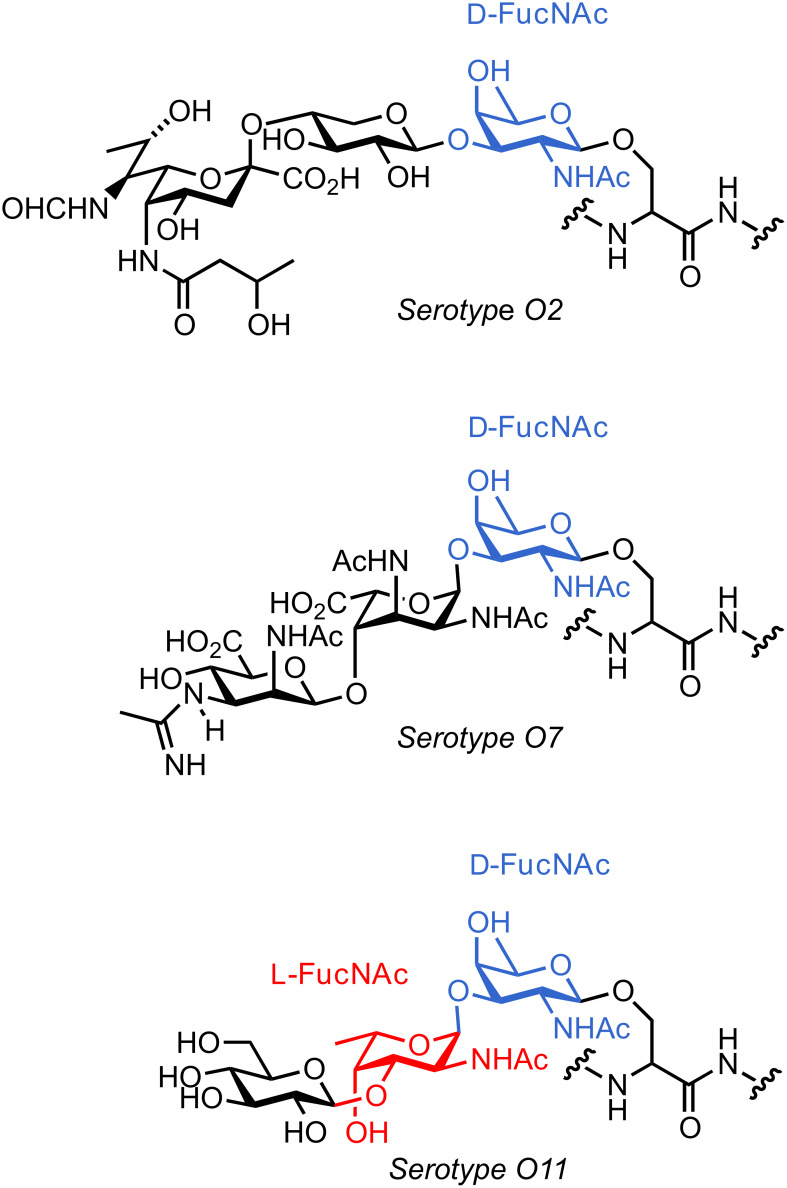
Structure of some *O*-linked glycans found on the cell surface of *P. aeruginosa*.

Our efforts were directed to the development of new vaccine candidates [14–16] to prevent bacterial infections, including glycans of the highly pathogenic bacteria *Pseudomonas aeruginosa*. *P. aeruginosa* is a nosocomial pathogen that is involved in ventilator-associated pneumonia and has become resistant to many antimicrobials. The somatic pili of *P. aeruginosa* are a major virulence factor playing a pivotal role in the adherence and invasiveness of the bacterium. In 2001, the *P. aeruginosa* pilin *O*-linked glycans were found to be linear trisaccharides that are covalently attached to serine (Figure 1). The *O*-glycans contain a D-fucosamine residue at the protein-binding site. This unusual monosaccharide is not present in eukaryotes, and therefore may be used to stimulate an antibacterial response in the host organism [17–23]. Access to differentially protected D- and L-fucosamine building blocks, which can be used in preparing the corresponding glycans, is instrumental for the evaluation of oligosaccharide-based vaccine candidates against this bacterium [24–28].

The synthesis of fucosamine building blocks has been reported in the literature, but it is highly affected by long synthetic sequences, extensive protecting group manipulations and expensive starting materials [29–33]. The *de novo* synthesis of rare sugars [34–43] provides an attractive alternative for rapid access to the required building blocks, but this approach has not been reported for D- or L-fucosamine [29–33,44–46].

Here, a full account of the *de novo* synthesis of differentially protected D- and L-fucosamine building blocks is described following a recent preliminary communication [47]. The building blocks prepared by *de novo* synthesis were used in the assembly of two disaccharides that are found on *P. aeruginosa*.

## Results and Discussion

### *De novo* synthesis of D- and L-fucosamine building blocks

**Scheme 1:**
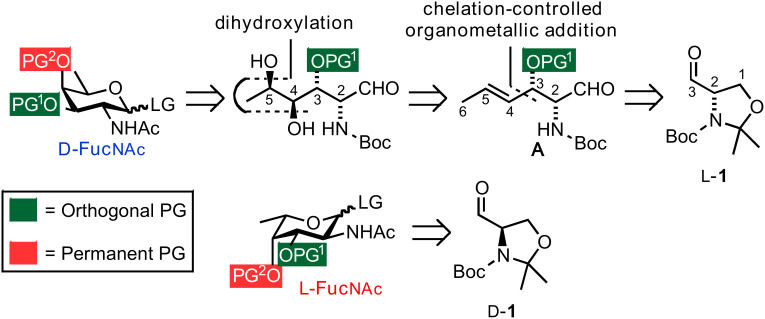
Retrosynthetic analysis of D- and L-fucosamine building blocks.

Our retrosynthetic analysis of D-fucosamine envisioned the installation of the *syn*-1,2-diol unit by osmium-catalysed dihydroxylation of allylic ether **A**. It was anticipated that the conformation adopted by the molecule would allow for the formation of the required *anti* relationship between *C*3 and *C*4 hydroxy groups. **A** in turn would be accessed by the addition of a carbon nucleophile to L-Garner aldehyde L-**1** (Scheme 1) [48]. The fact that D- and L-Garner aldehydes are commercially available greatly facilitates the synthesis of both D- and L-fucosamine building blocks. The fucosamine residues are usually further elongated at the *C*3 position in *P. aeruginosa* requiring two orthogonal protecting groups (PGs) at *C*3 and *C*4 of the building block in order to differentiate the two hydroxy groups at a later stage.

**Scheme 2:**
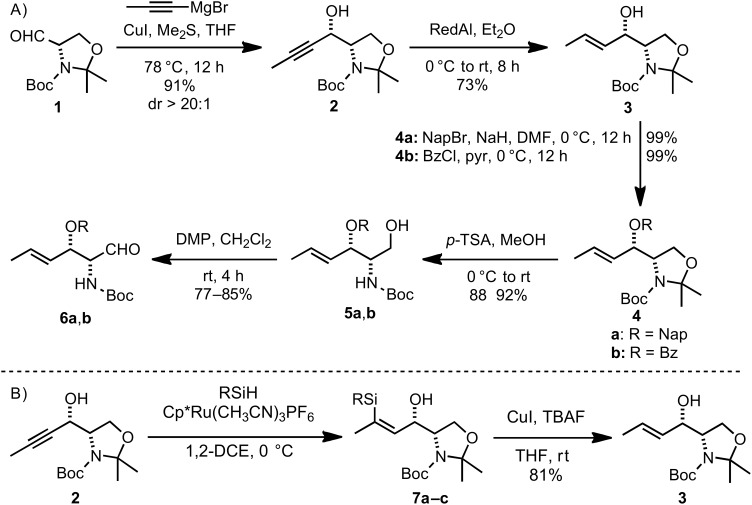
(A) Synthesis of aldehydes **6a** and **6b**. (B) Alkyne reduction by hydrosilylation–protodesilylation sequence (see Table 1).

The three carbon *C*4–*C*6 fragment of D-fucosamine was introduced by chelation-controlled addition [49–50] of commercially available propynylmagnesium bromide to L-**1** (Scheme 2A) [51–52] The following *E*-selective alkyne reduction was accomplished by using diluted RedAl in Et_2_O [53]. This reaction proved to be highly dependent on the quality of the aluminium reagent obtained from commercial sources. Inspired by the work from the Trost group [54–55], an alternative reduction protocol based on a Ru-catalyzed hydrosilylation–protodesilylation sequence was pursued (Scheme 2B). Thus, exposure of alkyne **2** to Cp*Ru(CH_3_CN)_3_PF_6_ catalyst in the presence of the appropriate trialkylsilane gave the desired silylated products **7a**–**c** (Table 1). The alkene geometry was confirmed as *Z* by nuclear Overauser effect (nOe). Treatment of **7a**–**c** with TBAF and CuI delivered **3** in high yields. Generally, BnMe_2_SiH has been found to be optimal for these transformations [54–55]. In our hands however, the best results were achieved using the cheaper Et_3_SiH (Table 1). With a gram amount of **3** in hand, the *C*3 hydroxy group was protected and the acetonide removed by treatment with *p-*TSA. Oxidation of primary aminoalcohols **5a**–**b** with the Dess–Martin reagent [56] yielded the desired aldehydes **6a**–**b**, in five steps from commercially available starting materials. The two different *O*-protecting groups, namely naphthyl ether (Nap) [57–59] and benzoate ester (Bz), were introduced to gain access to two sets of electronically different and orthogonally protected derivatives (Scheme 2A).

**Table 1:** Hydrosilylation yields.

entry		R	yield (%)

1	**7a**:	Et_3_	81
2	**7b**:	Et_2_Me	71
3	**7c**:	Me_2_Bn	traces

**Scheme 3:**
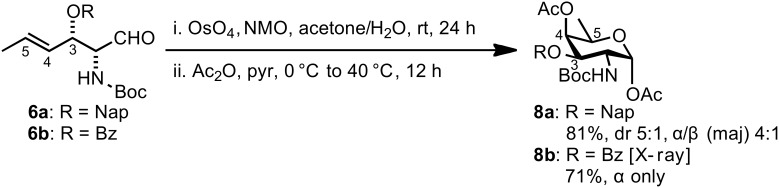
Synthesis of D-fucosamine building blocks **8a** and **8b**.

Dihydroxylation of aldehyde **6a** under standard Upjohn conditions gave, after peracetylation with Ac_2_O, D-fucosamine building block D-**8a** in 81% yield and 5:1 dr (*anti*/*syn*, diastereomers separable by column chromatography) (Scheme 3) [60–62]. The formation of the desired *C*3–*C*4–*C*5 *syn*,*syn* cyclic product was confirmed based on observation of a ^3^*J_H_*_3–_*_H_*_4_ coupling of 3.5 Hz. When the same sequence of dihydroxylation–peracetylation was performed on Bz-substituted aldehyde **6b**, compound D-**8b** was formed in 71% as a single diastereomer (Scheme 3). This product was crystallized from *n*-hexane/EtOAc solvent mixture, and the stereochemical assignment was confirmed by X-ray analysis (data shown in Supporting Information File 1).

**Scheme 4:**
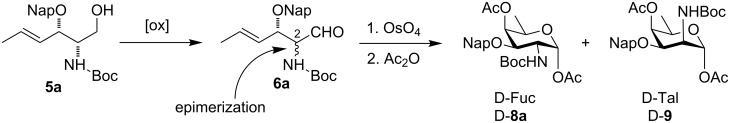
Epimerization of aldehyde **6a**.

During the optimization of the synthetic sequence we explored potentially more economic oxidation protocols to convert the primary alcohol **5a** to the D-fucosamine building block. Since α-amino protected aldehydes easily undergo α-epimerization, oxidation had to proceed under mild conditions in order to avoid the formation of the undesired D-talosamine building block D-**9** (Scheme 4).

Several oxidation methods were tested (Table 2). All the reactions were performed sequentially without column-chromatographic purification of the aldehyde. Classic Swern oxidation [63] (Table 2, entry 2) gave a 2:1 mixture of *C*2 epimers demonstrating the acid lability of aldehyde **6a**. This ratio in favour of the desired product was improved to 8:1 by switching the base from Et_3_N to iPr_2_EtN as reported by Dondoni and co-workers (Table 2, entry 3) [64]. Parrikh–Doering oxidation [65] and TCCA–TEMPO mediated oxidation [66] (Table 2, entries 4 and 6, respectively) were not suitable as considerable amounts of D-talosamine building block were formed. DMP emerged as the reagent of choice for the oxidation of **5a**. Interestingly, the dihydroxylation of aldehyde *epi*-**6a** resulted in the formation of talosamine D-**9** as a single diastereomer while the dihydroxylation of **6a** gave D-**8a** in 5:1 dr (Scheme 3).

**Table 2:** Oxidation of **5a** to D-fucosamine D-**8a**.

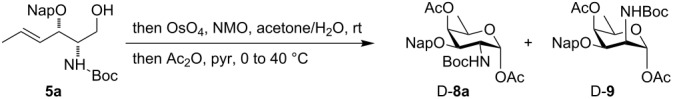		

entry	reaction conditions	D-Fuc/D-Tal^a^

1^b^	DMP, CH_2_Cl_2_, rt then 1 M Na_2_S_2_O_3_ in NaHCO_3_, sat.	>20:1
2	DMSO, (COCl)_2_, Et_3_N, CH_2_Cl_2_, −78 °C	2:1
3	DMSO, (COCl)_2_, iPr_2_NEt, CH_2_Cl_2_, −78 °C	8:1
4	SO_3_·pyr, DMSO, CH_2_Cl_2_, 0 °C	2:1
5	TCCA, TEMPO, CH_2_Cl_2_, 0 °C	3:1

^a^Determined by ^1^H NMR analysis of the crude product after dihydroxylation and peracetylation. ^b^Reaction run on 1 g scale.

**Scheme 5:**
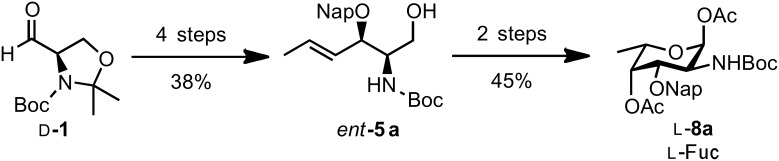
Synthesis of L-fucosamine building block L-**8a** from D-Garner aldehyde.

Carrying out the synthetic sequence optimized for the synthesis of D-**8a**, on D-Garner aldehyde (D-**1**, commercially available) gave alcohol *ent*-**5a** (four steps, two chromatographic purifications) that after the direct oxidation–dihydroxylation–peracetylation protocol yielded the desired L-fucosamine building block L-**8a** (Scheme 5).

### Synthesis of fucosamine-containing disaccharides

**Scheme 6:**
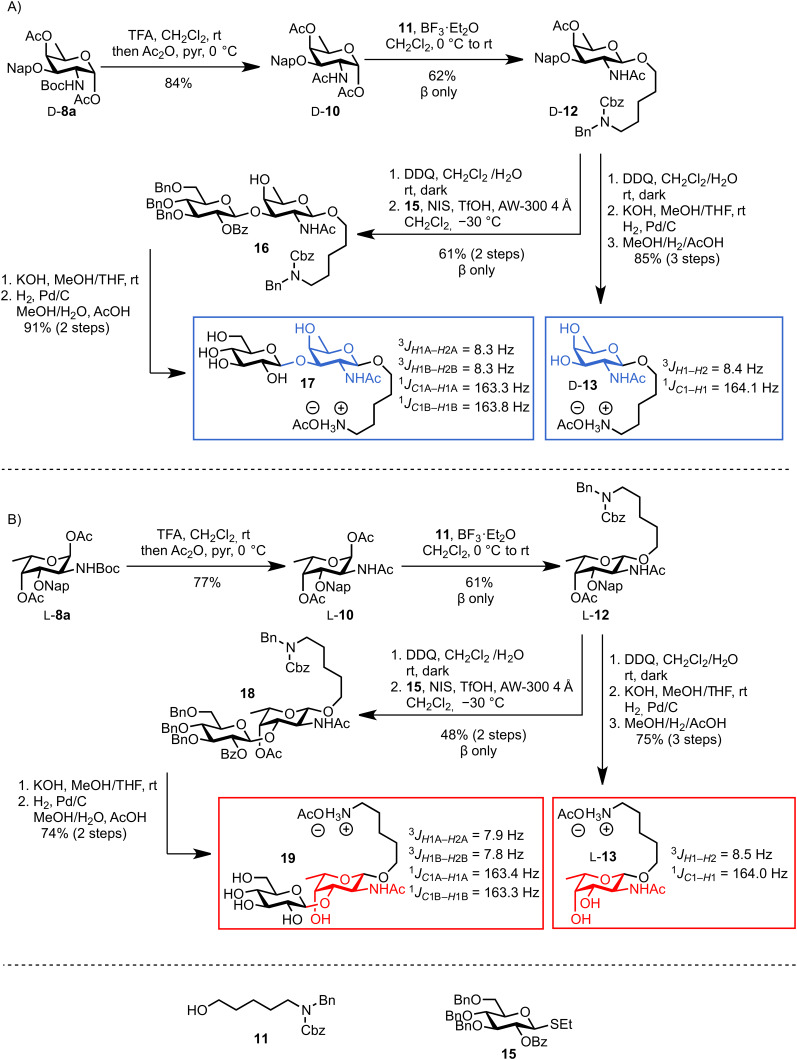
Synthesis of D- and L-fucosamine-containing mono- and disaccharides carrying the pentanolamine linker.

To facilitate the use of the bacterial monosaccharides in the glycan microarray platform and their conjugation to carrier proteins, an appropriate linker was required at the anomeric position [67]. Placement of a *C*3 naphthyl ether anticipated the site for further glycosylation at this position, which typically serves as the connection to the next sugar. Therefore, building block **8a** is ideal in terms of orthogonality and chemical synthesis. Hence, we tested the ability of D-**8a** to undergo anomeric functionalization and to effect glycosylation at the *C*3 hydroxy group. Due to the presence of *N*-acetylated D- and L-fucosamine residues in *P. aeruginosa O*-linked glycans, the strategic *N*-protecting group was evaluated. Direct glycosylation by using the building block D-**8a** was not possible. Hence, a direct *N*-Boc deprotection/*N*-acetylation sequence afforded D-**10** in 84% yield (Scheme 6A). Direct glycosylation of D-**10** by using glycosylating agent **11** [67] and BF_3_·Et_2_O as the activating agent, yielded the linker-functionalized monosaccharide D-**12** as the β-anomer (^3^*J_H_*_1–_*_H_*_2_ = 8.3 Hz, Scheme 6A). At this point, the *C*3 naphthyl ether was cleaved under oxidative conditions and the corresponding alcohol was revealed by using a two-step deprotection protocol consisting of ester hydrolysis and hydrogenation. Monosaccharide D-**13** was obtained in 85% yield (the β-linkage further confirmed by ^1^*J_C_*_1–_*_H_*_1_ 164.1 Hz, Scheme 6A). Direct glycosylation after DDQ-deprotection was possible and the use of differentially protected glucose building block **15** [68] yielded the desired β-disaccharide **16** in 61% yield over two steps (β anomer, ^3^*J_H_*_1–_*_H_*_2_ of 7.8 Hz, Scheme 6A). Global deprotection employed saponification, and hydrogenation gave the fully deprotected D-fucosamine containing disaccharide **17** (Scheme 6A).

When the same synthetic sequence was performed on L-FucNAc building block L-**10** the fully deprotected monomer L-**13** and disaccharide **19** were obtained in similar yields and selectivities (Scheme 6B). Diagnostic ^1^*J_C_*_–_*_H_* analysis also corroborated the formation of the β-glycosydic linkages (Scheme 6B). Linker-terminated disaccharide **19** represents the terminal unit of the *P. aeruginosa* serotype *O*11 *O*-linked glycan (Figure 1).

## Conclusion

The *de novo* synthesis of differentially protected D- and L-fucosamine building blocks from D- and L-Garner aldehyde is reported. Placement of a naphthyl ether protecting group at the *C*3 position allows for further elongation by glycosylation. The key oxidation step was optimized to minimize the formation of the unwanted D-talosamine building block D-**9**. The fucosamine building blocks prepared by *de novo* synthesis enabled the preparation of monosaccharides and disaccharides for attachment to microarray surfaces. The terminal disaccharide of *P. aeruginosa O*11 *O*-linked glycan has been prepared and will be the basis for biological studies involving this pathogen.

## Experimental

### General experimental details

All reagents were obtained from commercial suppliers and were used without further purification unless otherwise specified. All reactions were conducted under an Ar atmosphere by using standard Schlenk techniques. THF and Et_2_O were distilled from purple Na/benzophenone diketyl; CH_2_Cl_2_, pyridine and BF_3_·Et_2_O were distilled from CaH_2_. Deionized water was obtained from an in-house purification system. The compounds purified by flash chromatography were further concentrated by the removal of residual solvent under high vacuum (<0.2 mbar). ^1^H NMR and ^13^C NMR spectra were measured with a Varian 400-MR or Varian 600 spectrometer. The proton signal of residual, nondeuterated solvent (δ 7.26 ppm for CHCl_3_ or δ 4.79 ppm for HDO) was used as an internal reference for ^1^H spectra. For ^13^C spectra, the chemical shifts are reported relative to the δ 77.36 ppm resonance of CDCl_3_. Coupling constants (*J* values) are quoted to one decimal place with values in hertz (Hz) and were corrected. Infrared (IR) spectra were recorded as thin films on a Perkin Elmer Spectrum 100 FTIR spectrophotometer. Optical rotations (OR) were measured with a Schmidt & Haensch UniPol L 1000 spectrometer at a concentration (*c*) expressed in grams per hundred millilitres (g/100 mL). High-resolution mass spectra (HRMS) were recorded with an Agilent 6210 ESI–TOF mass spectrometer at the Freie Universität Berlin, Mass Spectrometry Core Facility. Analytical thin-layer chromatography (TLC) was performed on Kieselgel 60 F_254_ glass plates precoated with a 0.25 mm thickness of silica gel. The TLC plates were visualized with UV light and by staining with Hanessian solution (ceric sulfate and ammonium molybdate in aqueous sulfuric acid) or potassium permanganate solution (potassium permanganate in basic aqueous solution). Column chromatography was performed by using Kieselgel 60 (230–400 mesh) silica gel with a typical 50–100:1 weight ratio of silica gel to crude product. For the preparation and characterization of compounds **2**–**10**, D-**12** and D-**16** see [14].

### L-Fucosamine monosaccharide L-**12**

Under the same reaction conditions reported in [14], L-**10** (75 mg, 0.17 mmol, 1.0 equiv), **11** (86 mg, 0.26 mmol, 1.5 equiv) and BF_3_·Et_2_O (33 mL, 0.26 mmol, 1.5 equiv) gave L-**12** (74 mg, 61%) as an oil; [α]_D_^20^ −63.2 (*c* 2.0, CHCl_3_), other data as above.

### D-Fucosamine monosaccharide D-**13**


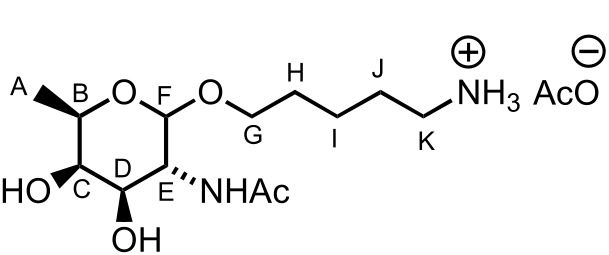


A solution of D-**12** (56 mg, 80.0 mmol, 1.0 equiv) in CH_2_Cl_2_ (0.9 mL) and H_2_O (90 mL) was treated with DDQ (21 mg, 94.0 mmol, 1.2 equiv) and stirred in the dark at rt for 2 h. Saturated NaHCO_3_ (2 mL) and CH_2_Cl_2_ (5 mL) were added and the layers were separated. The aqueous layer was extracted with CH_2_Cl_2_ (2 × 3 mL). The combined organic layers were dried (MgSO_4_), filtered and concentrated. The crude was purified by filtration over a short plug of silica gel eluting with CH_2_Cl_2_/MeOH 9:1 (34 mg, 76%) and immediately used for the next step. The monodeprotected compound (18 mg, 32.0 mmol, 1.0 equiv) in MeOH (0.4 mL) and THF (0.4 mL) was treated with KOH (1.8 mg, 32.0 mmol, 1.0 equiv) and stirred at rt for 30 min. H_2_O (2 mL) was added and the solvents were removed under vacuum. CH_2_Cl_2_ (5 mL) was added and the layers were separated. The aqueous layer was extracted with CH_2_Cl_2_ (2 × 5 mL). The combined organic layers were dried (MgSO_4_), filtered and concentrated. The crude product was solubilised in MeOH/H_2_O/AcOH (2.0:1.0:0.05 mL) and Pd/C (10 mg) was added. The heterogeneous mixture was stirred under an atmosphere of H_2_ for 24 h. The mixture was filtered over celite to give D-**13** (8 mg, 85%) as an amorphous solid; [α]_D_^20^ +112.9 (*c* 1.1, H_2_O); ^1^H NMR (400 MHz, D_2_O) δ 4.38 (d, *J* = 8.4 Hz, 1H, CH^F^), 3.83 (dt, *J* = 10.1, 6.3 Hz, 1H, CH^G^), 3.79 (dd, *J* = 10.7, 8.4 Hz, 1H, CH^E^), 3.75–3.69 (m, 2H, CH^C^ and NH), 3.68 (dd, *J* = 10.7, 3.4 Hz, 1H, CH^D^), 3.66 (dt, *J* = 10.1, 6.3 Hz, 1H, CH^G^), 3.41 (q, *J* = 6.4 Hz, 1H, CH^B^), 2.95 (t, *J* = 7.6 Hz, 2H, CH_2_^K^), 1.99 (s, 3H, CH_3_), 1.87 (AcOH), 1.71–1.62 (m, 2H, CH_2_^J^), 1.59–1.51 (m, 2H, CH_2_^H^), 1.39–1.29 (m, 2H, CH_2_^I^), 1.23 (d, *J* = 6.4 Hz, 3H, CH_3_^A^); ^13^C NMR (100 MHz, D_2_O) δ 101.4 (CH^F^), 71.0 (CH^D^), 70.4 (CH^C^), 70.3 (CH^B^), 69.8 (CH_2_^G^), 52.0 (CH^E^), 39.2 (CH_2_^K^), 28.0 (CH_2_^J^), 26.3 (CH_2_^H^), 23.1 (CH_3_), 22.1 (CH_3_), 21.9 (CH_2_^I^), 15.3 (CH_3_^A^); LRMS–ESI (*m*/*z*): 291.2 [M + H^+^]; HRMS–ESI (*m*/*z*): [M + H^+^] calcd for C_13_H_27_N_2_O_5_, 291.1914; found, 291.1919.

### L-Fucosamine monosaccharide L-**13**

Using the same reaction conditions reported for D-**13**, D-**12** (24 mg, 34.0 mmol, 1.0 equiv), DDQ (21 mg, 41.0 mmol, 1.2 equiv), KOH (1.8 mg, 34.0 mmol, 1.0 equiv) and Pd/C (10 mg), gave L-**13** (9 mg, 75%) as an amorphous solid; [α]_D_^20^ −111.1 (*c* 1.0, H_2_O), other data as above.

### D-Fucosamine disaccharide **17**


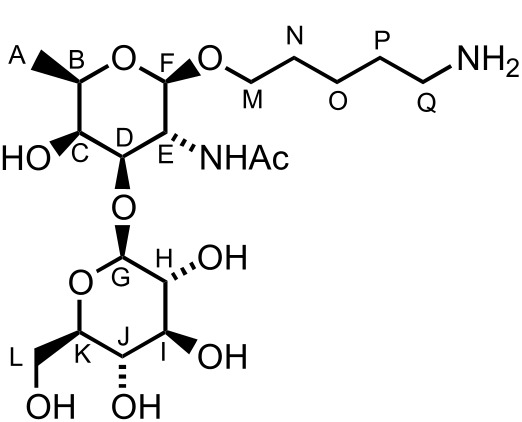


A solution of **16** (21 mg, 19.0 mmol, 1.0 equiv) in MeOH (0.4 mL) and THF (0.4 mL) was treated with KOH (1.0 mg, 19.0 mmol, 1.0 equiv) and stirred at rt for 30 min. H_2_O (2 mL) was added and the solvents were removed under vacuum. CH_2_Cl_2_ (5 mL) was added and the layers were separated. The aqueous layer was extracted with CH_2_Cl_2_ (2 × 5 mL). The combined organic layers were dried (MgSO_4_), filtered and concentrated. The crude product was solubilised in MeOH/H_2_O/AcOH (2.0:1.0:0.05 mL) and Pd/C (20 mg) was added. The heterogeneous mixture was stirred under an atmosphere of H_2_ for 24 h. The mixture was filtered over celite to give **17** (9 mg, 91%) as an amorphous solid; [α]_D_^20^ +13.5 (*c* 0.9, H_2_O); ^1^H NMR (400 MHz, D_2_O) δ 4.47 (d, *J* = 7.9 Hz, 1H, CH^G^), 4.41 (d, *J* = 7.9 Hz, 1H, CH^F^), 3.94 (d, *J* = 2.7 Hz, 1H, CH^C^), 3.90 (dd, *J* = 10.3, 7.9 Hz, 1H, CH^E^), 3.87–3.77 (m, 3H, CH^L^ & CH^M^ & CH^D^), 3.70 (q, *J* = 6.5 Hz, 1H, CH^B^), 3.68 (dd, *J* = 12.1, 3.4 Hz, 1H, CH^L^), 3.54 (dt, *J* = 10.0, 6.2 Hz, 1H, CH^M^), 3.44–3.32 (m, 3H, CH^I^, CH^J^ and CH^K^), 3.25 (t, *J* = 7.9 Hz, 1H, CH^H^), 2.94 (t, *J* = 7.6 Hz, 2H, CH_2_^Q^), 1.97 (s, 3H, CH_3_), 1.90 (s, 3H, AcOH), 1.68–1.59 (m, 2H, CH_2_^P^), 1.58–1.50 (m, 2H, CH_2_^N^), 1.41–1.32 (m, 2H, CH_2_^O^), 1.22 (d, *J* = 6.5 Hz, 3H, CH_3_^A^); ^13^C NMR (100 MHz, D_2_O) δ 104.2 (CH^G^), 101.1 (CH^F^), 80.2 (CH^D^), 75.5 (CH), 75.4 (CH), 72.7 (CH^H^), 70.4 (2 × CH^B&C^), 69.8 (CH_2_^L^), 69.2 (CH), 60.3 (CH_2_^M^), 50.8 (CH^E^), 39.1 (CH_2_^Q^), 28.0 (CH_2_^N^), 26.2 (CH_2_^P^), 23.1 (CH_3_), 22.1 (CH_3_), 22.0 (CH_2_^O^), 15.3 (CH_3_^A^); LRMS–ESI (*m*/*z*): 453.2 [M + Na^+^]; HRMS–ESI (*m*/*z*): [M + H^+^] calcd for C_19_H_37_N_2_O_10_, 453.2443; found, 453.2468.

### L-Fucosamine disaccharide **18**


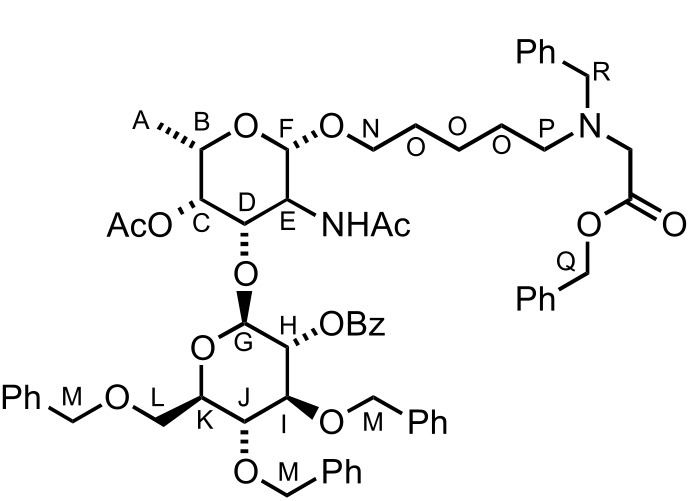


A solution of L-**12** (60 mg, 86 mmol, 1.0 equiv) in CH_2_Cl_2_ (1 mL) and H_2_O (0.1 mL) was treated with DDQ (21 mg, 95 mmol, 1.2 equiv) and stirred in the dark at rt for 2 h. Saturated NaHCO_3_ (2 mL) and CH_2_Cl_2_ (5 mL) were added and the layers were separated. The aqueous layer was extracted with CH_2_Cl_2_ (2 × 3 mL). The combined organic layers were dried (MgSO_4_), filtered and concentrated. The crude was purified by filtration over a short plug of silica gel eluting with CH_2_Cl_2_/MeOH (9:1) and immediately used for the next step. An oven-dried flask was immediately charged with the product and **15** (77 mg, 0.12 mmol, 1.5 equiv) was added. The flask was left under high vacuum for 4 h. After refilling the flask with argon, freshly distilled CH_2_Cl_2_ (1.5 mL) was added, and the mixture was cooled to −30 °C. NIS (29 mg, 0.13 mmol, 1.5 equiv) and TfOH (4 mL, 43 mmol, 0.5 equiv) were added. The mixture was stirred at −30 °C for 4 h and then it was allowed to warm to rt overnight. Saturated Na_2_SO_3_ (3 mL) and CH_2_Cl_2_ (3 mL) were added and the layers were separated. The aqueous layer was extracted with CH_2_Cl_2_ (2 × 5 mL). The combined organic layers were dried (MgSO_4_), filtered and evaporated. Purification by column chromatography on silica gel, eluting with *n*-hexane/EtOAc (6:4), gave **18** (45 mg, 48%) as an oil; *R*_f_ 0.22 [*n*-hexane/EtOAc (4:1)]; [α]_D_^20^ −5.5 (*c* 0.3, CHCl_3_); FTIR (film) ν_max_ (cm^−1^): 3321, 2925, 2857, 1743, 1716, 1705, 1662, 1368, 1271, 1240, 1071; ^1^H NMR (400 MHz, CDCl_3_, rotamers) δ 8.04 (d, 2H, *J* = 7.3 Hz, 2 × CH), 7.57 (tt, *J* = 7.4, 1H, 1.2 CH), 7.44 (t, *J* = 7.7 Hz, 2H, 2 × CH), 7.35–7.22 (m, 17H, 17 × CH), 7.17–7.04 (m, 8H, 8 × CH), 5.88 (br d, *J* = 6.5 Hz, 0.5H, NH), 5.73 (br d, *J* = 6.0 Hz, 0.5H, NH), 5.21 (dd, *J* = 9.1, 8.0 Hz, 1H, CH^H^), 5.17 (br s, 1H, CH^Q^), 5.14 (br s, 1H, CH^Q^), 5.13 (d, *J* = 3.5 Hz, 1H, CH^C^), 5.07 (d, *J* = 8.0 Hz, 1H, CH^F^), 4.79 (d, *J* = 10.8 Hz, 1H, CH^M^), 4.70 (d, *J* = 11.0 Hz, 1H, CH^M^), 4.65–4.53 (m, 6H, CH^D^ & CH^G^ & 4 × CH^M^), 4.52–4.46 (m, 2H, CH_2_^R^), 3.86 (t, *J* = 9.3 Hz, 1H, CH^I^), 3.82–3.74 (m, 4H, CH^J^, CH_2_^L^, CH^N^), 3.67 (q, *J* = 6.4 Hz, 1H, CH^B^), 3.50 (br dt, *J* = 9.6, 2.3 Hz, 1H, CH^K^), 3.44–3.40 (m, 1H, CH^N^), 3.23–3.17 (m, 3H, CH^E^ and CH_2_^P^), 1.85 and 1.81 (s, 3H, CH_3_), 1.53–1.50 (m, 3H, 3 × CH^O^), 1.45 (s, 3H, CH_3_), 1.29–1.21 (m, 3H, 3 × CH^O^), 1.08 (d, *J* = 6.4 Hz, 3H, CH_3_^A^); ^13^C NMR (100 MHz, CDCl_3_, rotamers) δ 171.2 (C=O), 170.5 (C=O), 165.1 (C=O), 137.9 (2 × C), 137.7 (2 × C), 137.6 (C), 133.2 (2 × CH), 130.1 (2 × CH), 129.5 (C), 128.5 (2 × CH), 128.4 (4 × CH), 128.3 (4 × CH), 128.2 (4 × CH), 127.9 (2 × CH), 127.8 (4 × CH), 127.7 (CH), 127.6 (CH), 127.3 (CH), 127.2 (CH), 127.1 (CH), 99.6 (CH), 98.4 (CH), 82.9 (CH), 77.7 (CH), 75.2 (CH_2_), 75.0 (CH_2_), 74.7 (CH), 73.5 (CH), 73.4 (CH_2_), 72.5 (CH), 69.7 and 69.5 (CH_2_), 69.1 (CH), 68.9 (CH), 68.7 (CH_2_), 67.1 (CH_2_), 54.4 (CH), 50.4 and 50.1 (CH_2_), 47.1 and 46.1 (CH_2_), 29.7 and 29.1 (CH_2_), 27.8 and 27.4 (CH_2_), 23.5 (CH_3_), 23.1 (CH_2_), 19.9 (CH_3_), 16.4 (CH_3_); LRMS–ESI (*m*/*z*): 1115.4 [M + Na^+^].

### L-Fucosamine disaccharide **19**


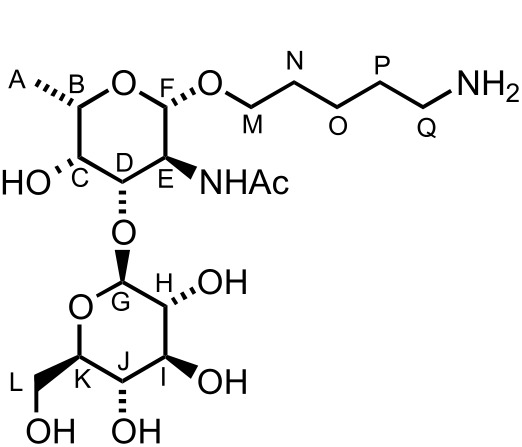


A solution of **18** (29 mg, 27.0 mmol, 1.0 equiv) in MeOH (0.6 mL) and THF (0.6 mL) was treated with KOH (0.8 mg, 13.5 mmol, 0.5 equiv) and stirred at rt for 30 min. H_2_O (2 mL) was added and the solvents were removed under vacuum. CH_2_Cl_2_ (5 mL) was added and the layers were separated. The aqueous layer was extracted with CH_2_Cl_2_ (2 × 5 mL). The combined organic layers were dried (MgSO_4_), filtered and concentrated. The crude product was solubilised in MeOH/H_2_O/AcOH (3.0:1.5:0.06 mL) and Pd/C (30 mg) was added. The heterogeneous mixture was stirred under an atmosphere of H_2_ for 24 h. The mixture was filtered over celite to give **19** (10 mg, 74%) as an amorphous solid; [α]_D_^20^ +59.2 (*c* 0.9, H_2_O); ^1^H NMR (400 MHz, D_2_O) δ 4.48 (d, *J* = 7.9 Hz, 1H, CH^G^), 4.43 (d, *J* = 7.9 Hz, 1H, CH^F^), 3.96–3.89 (m, 3H, CH^C^, CH^D^ and CH^L^), 3.88–3.80 (m, 2H, CH^E^ and CH^M^), 3.72 (q, *J* = 6.5 Hz, 1H, CH^B^), 3.67 (dd, *J* = 12.1, 6.5 Hz, 1H, CH^L^), 3.56 (dt, *J* = 10.2, 6.2 Hz, 1H, CH^M^), 3.45 (t, *J* = 9.5 Hz, 1H, CH^I^), 3.42–3.46 (m, 1H, CH^J^), 3.36–3.31 (m, 1H, CH^K^), 3.27 (dd, *J* = 9.5, 7.9 Hz, 1H, CH^H^), 2.95 (t, *J* = 7.6 Hz, 2H, CH_2_^Q^), 1.99 (s, 3H, CH_3_), 1.97 (s, 3H, AcOH), 1.69–1.60 (m, 2H, CH_2_^P^), 1.59–1.50 (m, 2H, CH_2_^N^), 1.42–1.32 (m, 2H, CH_2_^O^), 1.26 (d, *J* = 6.5 Hz, 3H, CH_3_^A^); ^13^C NMR (100 MHz, D_2_O) δ 177.0 (C=O), 103.9 (CH^F^), 102.7 (CH^G^), 80.1 (CH^D^), 79.5 (CH^I^), 77.9 (CH^J^), 75.3 (CH^B^), 73.0 (CH^H^), 72.4 (CH_2_^L^), 72.2 (CH^K^), 70.4 (CH^C^), 63.5 (CH_2_^M^), 52.8 (CH^E^), 41.7 (CH_2_^Q^), 30.5 (CH_2_^N^), 28.8 (CH_2_^P^), 24.8 (CH_3_), 24.5 (CH_2_^O^), 24.4 (CH_3_), 17.8 (CH_3_); LRMS–ESI (*m*/*z*): 453.2 [M + H^+^]; HRMS–ESI (*m*/*z*): [M + H^+^] calcd for C_19_H_37_N_2_O_10_, 453.244; found, 453.2468.

## Supporting Information

File 1 ^1^H NMR, COSY, ^13^C NMR and HSQC spectra and the crystallographic data file for D-**8b**.

## References

[1] Mariño K, Bones J, Kattla J J, Rudd P M (2010). Nat Chem Biol.

[2] Arnold J N, Wormald M R, Sim R B, Rudd P M, Dwek R A (2007). Annu Rev Immunol.

[3] Dube D H, Bertozzi C R (2005). Nat Rev Drug Discovery.

[4] Stallforth P, Lepenies B, Adibekian A, Seeberger P H (2009). J Med Chem.

[5] Dube D H, Champasa K, Wang B (2011). Chem Commun.

[6] Boltje T J, Buskas T, Boons G-J (2009). Nat Chem.

[7] Bieber D, Ramer S W, Wu C-Y, Murray W J, Tobe T, Fernandez R, Schoolnik G K (1998). Science.

[8] Banerjee A, Wang R, Supernavage S L, Ghosh S K, Parker J, Ganesh N F, Wang P G, Gulati S, Rice P A (2002). J Exp Med.

[9] Power P M, Ku S C, Rutter K, Warren M J, Limnios E A, Tapsall J W, Jennings M P (2007). Infect Immun.

[10] Takeuchi K, Taguchi F, Inagaki Y, Toyoda K, Shiraishi T, Ichinose Y (2003). J Bacteriol.

[11] Horzempa J, Carlson P E, O’Dee D M, Shanks R M Q, Nau G J (2008). BMC Microbiol.

[12] van Sorge N M, Bleumink N M C, van Vliet S J, Saeland E, van der Pol W-L, van Kooyk Y, van Putten J P M (2009). Cell Microbiol.

[13] Verma A, Arora S K, Kuravi S K, Ramphal R (2005). Infect Immun.

[14] Yang Y, Martin C E, Seeberger P H (2012). Chem Sci.

[15] Martin C E, Weishaupt M W, Seeberger P H (2011). Chem Commun.

[16] Ohara T, Adibekian A, Esposito D, Stallforth P, Seeberger P H (2010). Chem Commun.

[17] Smedley J G, Jewell E, Roguskie J, Horzempa J, Syboldt A, Stolz D B, Castric P (2005). Infect Immun.

[18] Castric P, Cassels F J, Carlson R W (2001). J Biol Chem.

[19] Chamot-Rooke J, Rousseau B, Lanternier F, Mikaty G, Mairey E, Malosse C, Bouchoux G, Pelicic V, Camoin L, Nassif X (2007). Proc Natl Acad Sci U S A.

[20] Kus J V, Kelly J, Tessier L, Harvey H, Cvitkovitch D G, Burrows L L (2008). J Bacteriol.

[21] Castric P (1995). Microbiology (Reading, U K).

[22] Sugawara K, Tsunakawa M, Konishi M, Kawaguchi J, Krishnan B, He C H, Clardy J (1987). J Org Chem.

[23] Myers A G, Liang J, Hammond M, Harrington P M, Wu Y, Kuo E Y (1998). J Am Chem Soc.

[24] Zhu X, Schmidt R R (2009). Angew Chem, Int Ed.

[25] Bongat A F G, Demchenko A V (2007). Carbohydr Res.

[26] Buskas T, Ingale S, Boons G-J (2006). Glycobiology.

[27] Codée J D C, Litjens R E J N, van den Bos L J, Overkleeft H S, van der Marel G A (2005). Chem Soc Rev.

[28] Boons G-J, Demchenko A V (2000). Chem Rev.

[29] Liav A, Hildesheim J, Zehavi U, Sharon N (1974). Carbohydr Res.

[30] Busca P, Martin O R (2004). Tetrahedron Lett.

[31] Jones G B, Lin Y, Xiao Z, Kappen L, Goldberg I H (2007). Bioorg Med Chem.

[32] Weerapana E, Glover K J, Chen M M, Imperiali B (2005). J Am Chem Soc.

[33] Amin M N, Ishiwata A, Ito Y (2006). Carbohydr Res.

[34] Schmidt R R (1987). Pure Appl Chem.

[35] Kirschning A, Jesberger M, Schöning K-U (2001). Synthesis.

[36] Hemeon I, Bennet A J (2007). Synthesis.

[37] Vogel P, Levy D E, Fugedi P (2006). The Organic Chemistry of Sugars.

[38] Voigt B, Scheffler U, Mahrwald R (2012). Chem Commun.

[39] Crich D, Navuluri C (2011). Org Lett.

[40] Lorpitthaya R, Suryawanshi S B, Wang S, Pasunooti K K, Cai S, Ma J, Liu X-W (2011). Angew Chem, Int Ed.

[41] Northrup A B, MacMillan D W C (2004). Science.

[42] Babu R S, Zhou M, O’Doherty G A (2004). J Am Chem Soc.

[43] Enders D, Grondal C (2005). Angew Chem, Int Ed.

[44] Calin O, Pragani R, Seeberger P H (2012). J Org Chem.

[45] Pragani R, Seeberger P H (2011). J Am Chem Soc.

[46] Pragani R, Stallforth P, Seeberger P H (2010). Org Lett.

[47] Leonori D, Seeberger P H (2012). Org Lett.

[48] Garner P (1984). Tetrahedron Lett.

[49] Cram D J, Kopecky K R (1959). J Am Chem Soc.

[50] O’Brien A G (2011). Tetrahedron.

[51] Zhang X, van der Donk W A (2007). J Am Chem Soc.

[52] Garner P, Park J M, Malecki E (1988). J Org Chem.

[53] Blot V, Jacquemard U, Reissig H-U, Kleuser B (2009). Synthesis.

[54] Trost B M, Ball Z T, Jöge T (2002). J Am Chem Soc.

[55] Trost B M, Ball Z T (2005). J Am Chem Soc.

[56] Dess D B, Martin J C (1983). J Org Chem.

[57] Gaunt M J, Yu J, Spencer J B (1998). J Org Chem.

[58] Liao W, Locke R D, Matta K L (2000). Chem Commun.

[59] Lipták A, Borbás A, Jánossy L, Szilágyi L (2000). Tetrahedron Lett.

[60] Cha J K, Kim N-S (1995). Chem Rev.

[61] Cha J K, Christ W J, Kishi Y (1983). Tetrahedron Lett.

[62] Karjalainen O K, Koskinen A M P (2011). Org Biomol Chem.

[63] Mancuso A J, Swern D (1981). Synthesis.

[64] Dondoni A, Perrone D (2004). Org Synth.

[65] Parikh J R, Doering W v E (1967). J Am Chem Soc.

[66] De Luca L, Giacomelli G, Masala S, Porcheddu A (2003). J Org Chem.

[67] Snapper C M, Mond J J (1996). J Immunol.

[68] Plé K, Chwalek M, Voutquenne-Nazabadioko L (2005). Tetrahedron.

