# Supplementary material for: De novo synthesis of D- and L-fucosamine containing disaccharides
Source: Beilstein J Org Chem. 2013 Feb 14;9:332–41. doi: 10.3762/bjoc.9.38 (PMC3596053; doi:10.3762/bjoc.9.38)
Supplement: File 1 — 1H NMR, COSY, 13C NMR and HSQC spectra and the crystallographic data file for D-8b. [file Beilstein_J_Org_Chem-09-332-s001.pdf]

Supporting Information

for

De novo synthesis of D- and L-fucosamine containing disaccharides

Daniele Leonori[1,§] and Peter H. Seeberger*[1,2]

Address: [1]Department of Biomolecular Systems, Max Planck Institute of Colloids and Interfaces, Am Mühlenberg 1, 14476 Potsdam, Germany and [2]Institute for Chemistry and Biochemistry, Freie Universität Berlin, Arnimallee 22, 14195 Berlin, Germany; [§]Current Address: School of Chemistry, University of Bristol, Cantock's Close, Bristol, BS8 1TS, UK.

Email: Peter H. Seeberger - peter.seeberger@mpikg.mpg.de

* Corresponding author

^{1}H NMR, COSY, ^{13}C NMR and HSQC spectra and the crystallographic data file for D-8b.

Table of Contents:

^{1}H, ^{13}C, COSY, HSQC spectra S2

X-ray structure of D-8b S15

^{1}H, ^{13}C, COSY and HSQC spectra

D-**13**, ^{1}H NMR, 400 MHz, D_2O

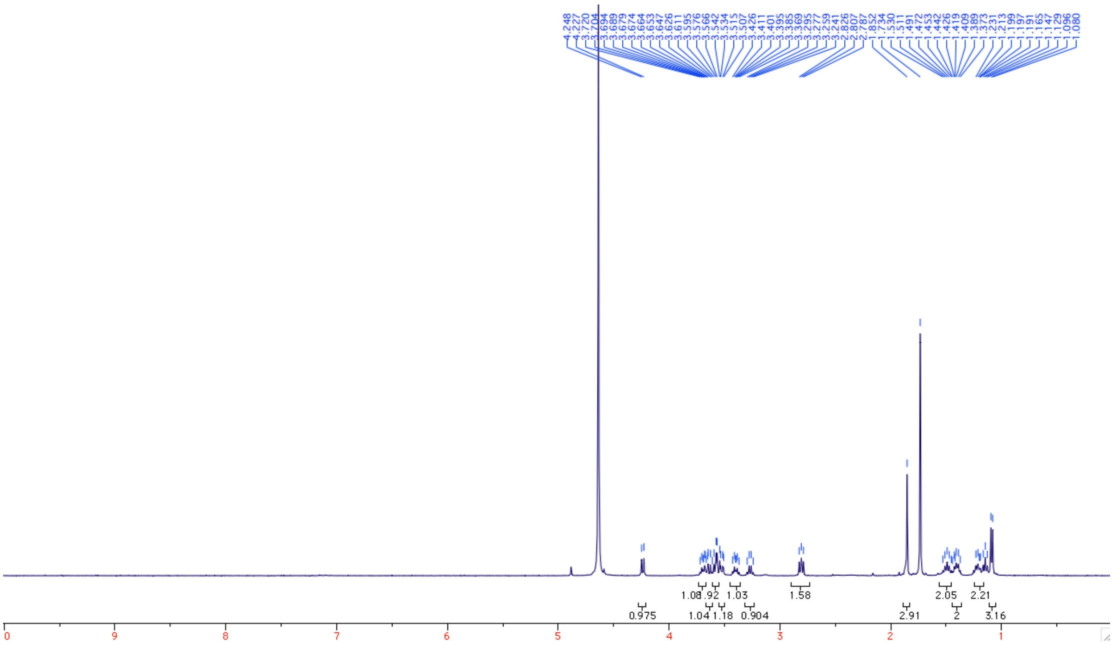

D-**13**, COSY, 400 MHz, D_2O

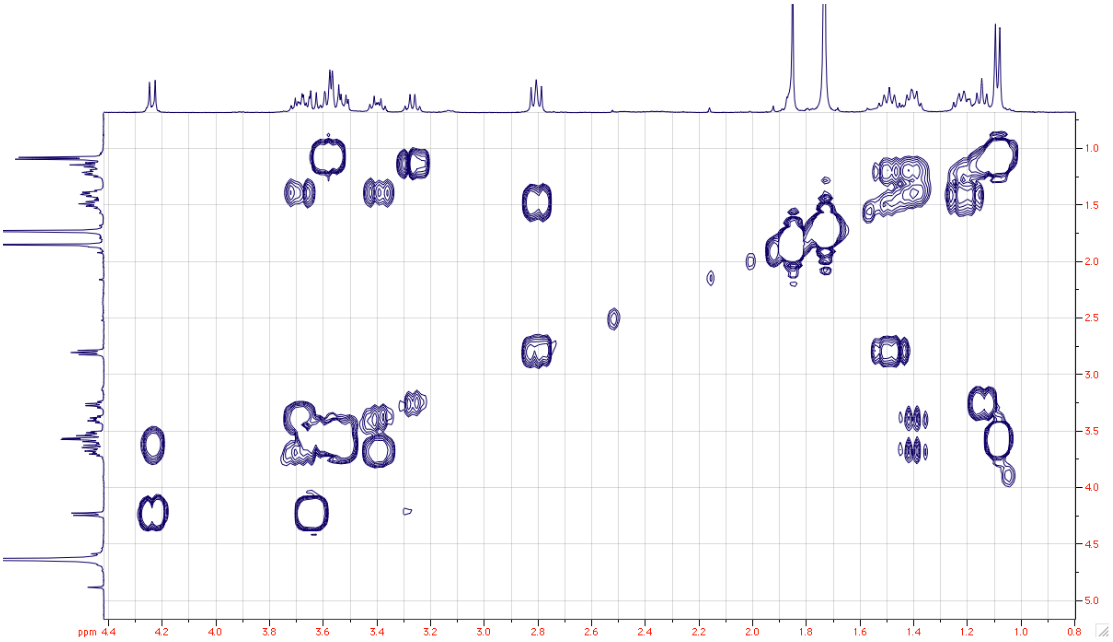

D-**13**, ^{13}C NMR, 100 MHz, D_2O

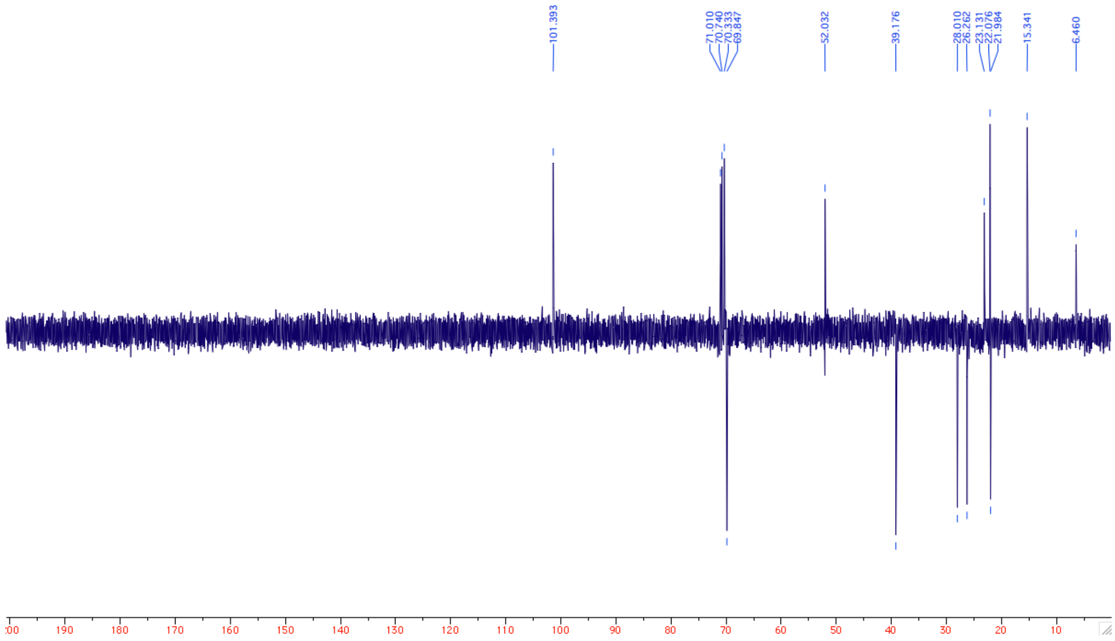

D-**13**, HSQC NMR, 400 MHz, D_2O

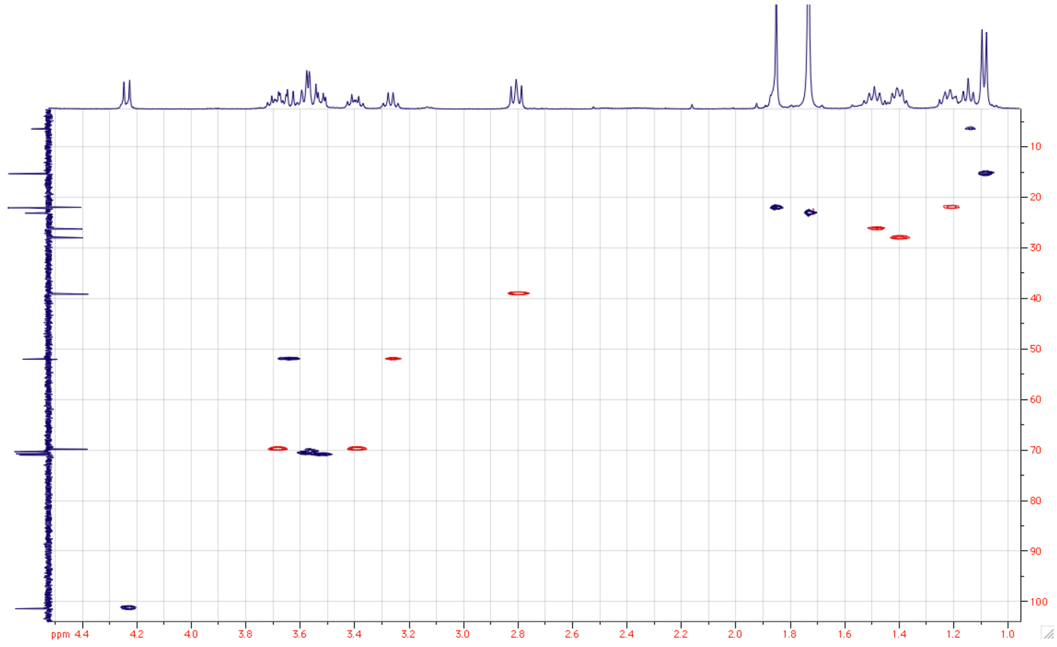

D-**13**, Coupled HSQC NMR, 400 MHz, D_2O

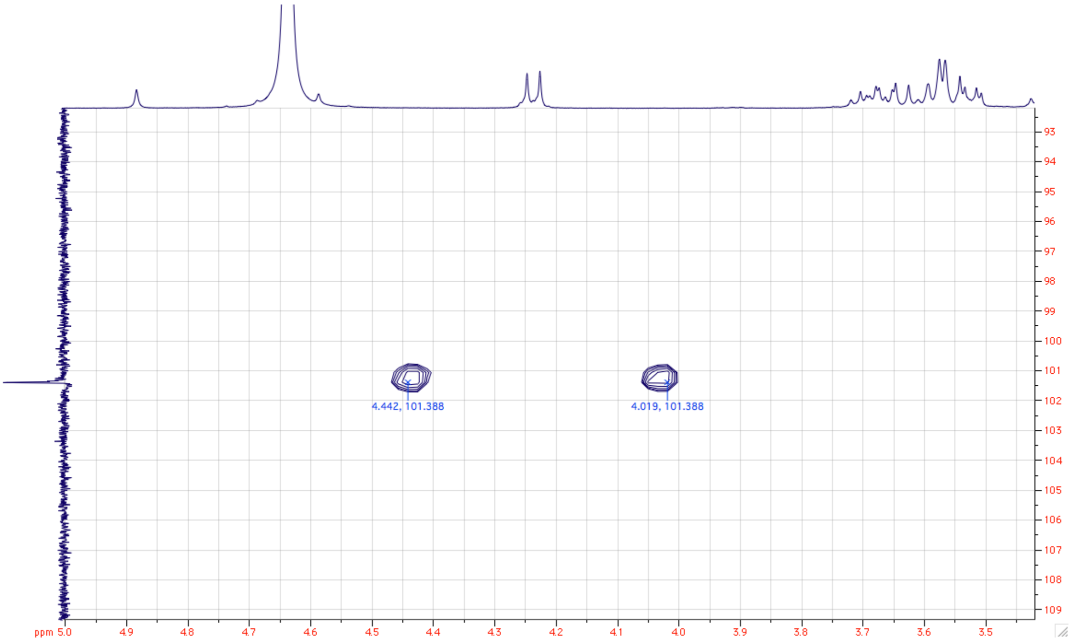

17, ^{1}H NMR, 400 MHz, D_2O

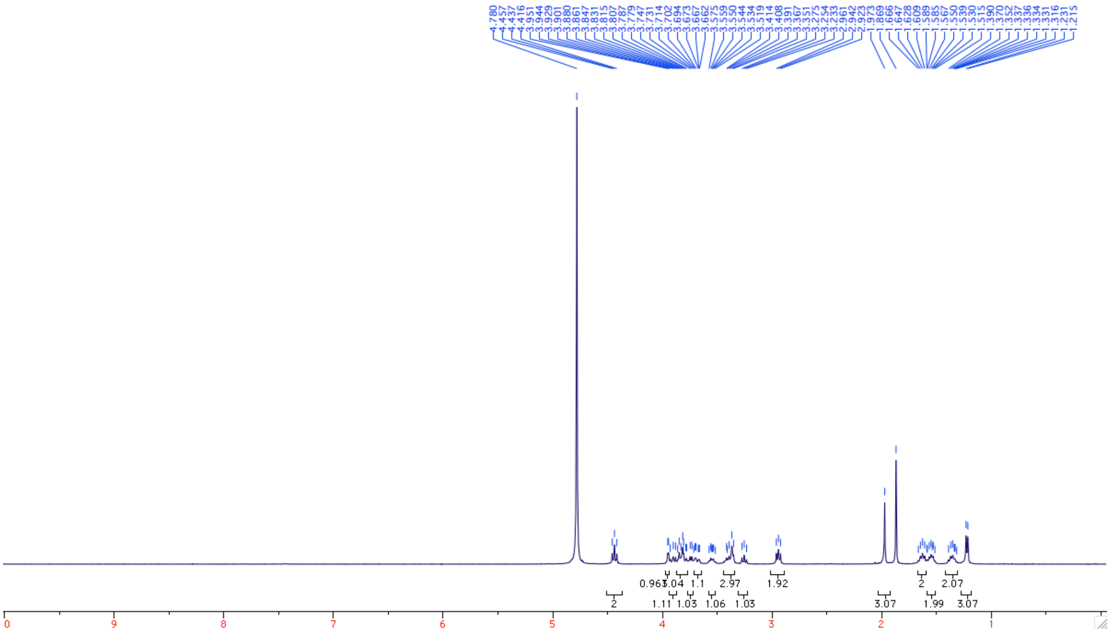

17, COSY NMR, 400 MHz, D_2O

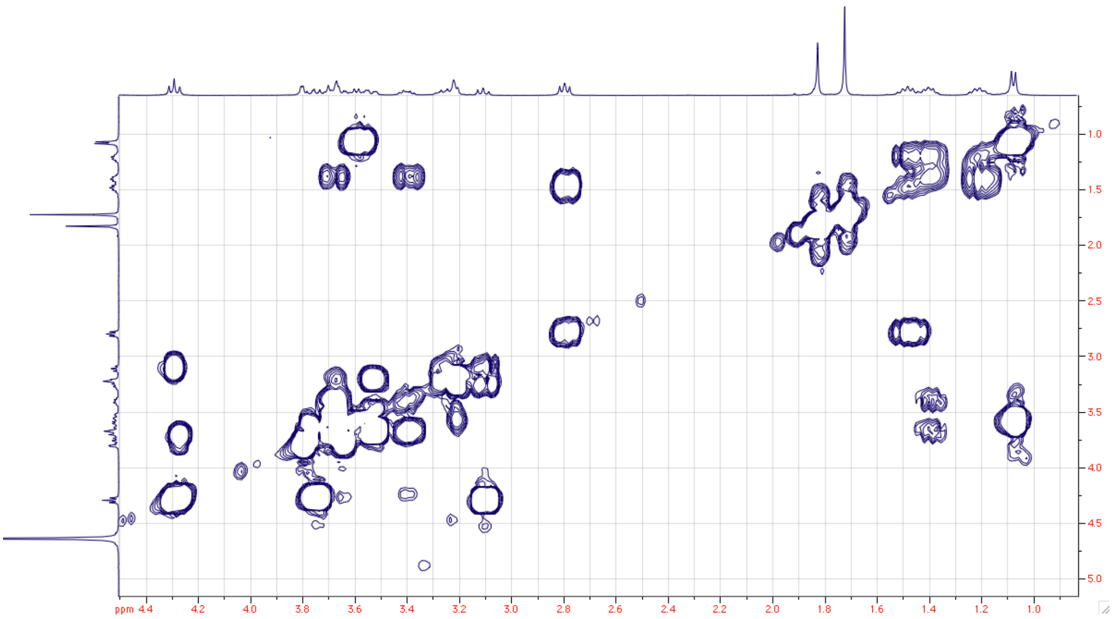

17, ^{13}C NMR, 400 MHz, D_2O

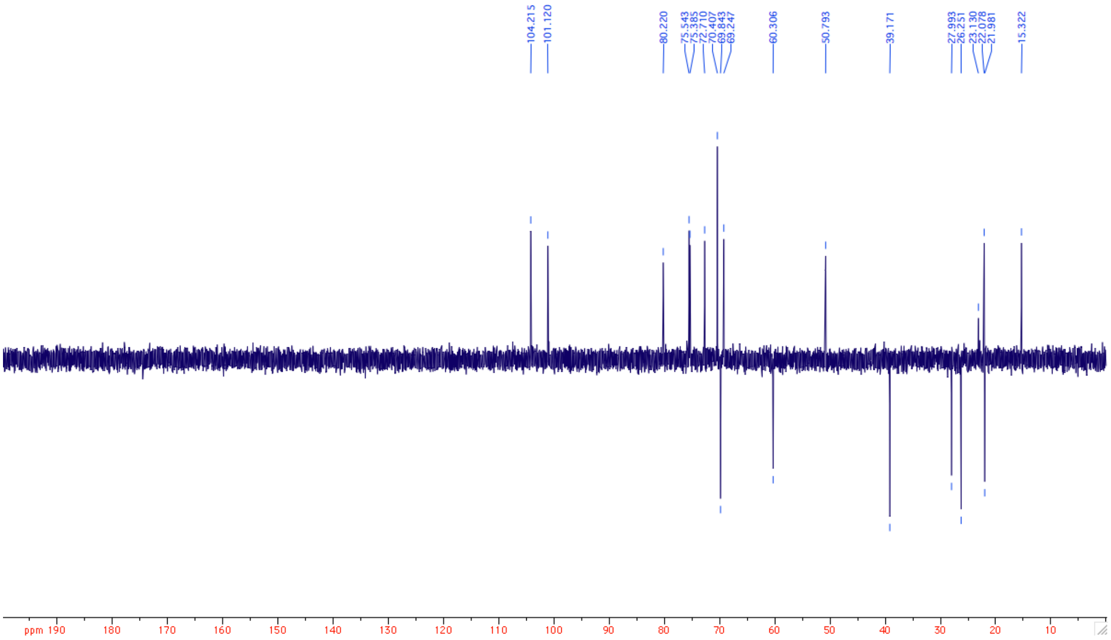

17, HSQC NMR, 400 MHz, D_2O

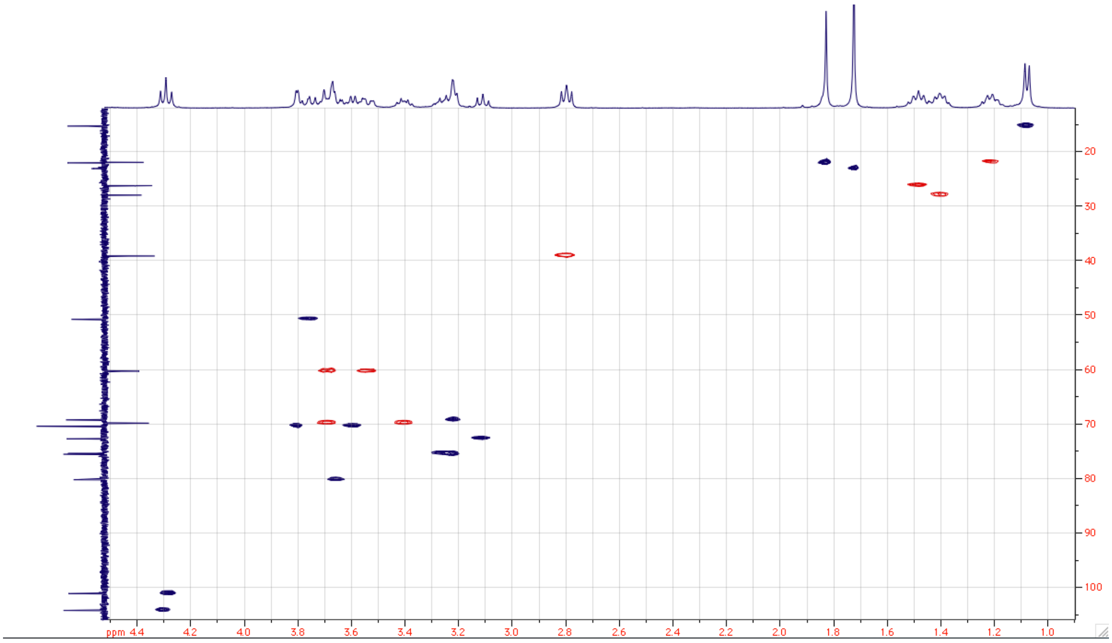

17, coupled HSQC, 400 MHz, D_2O

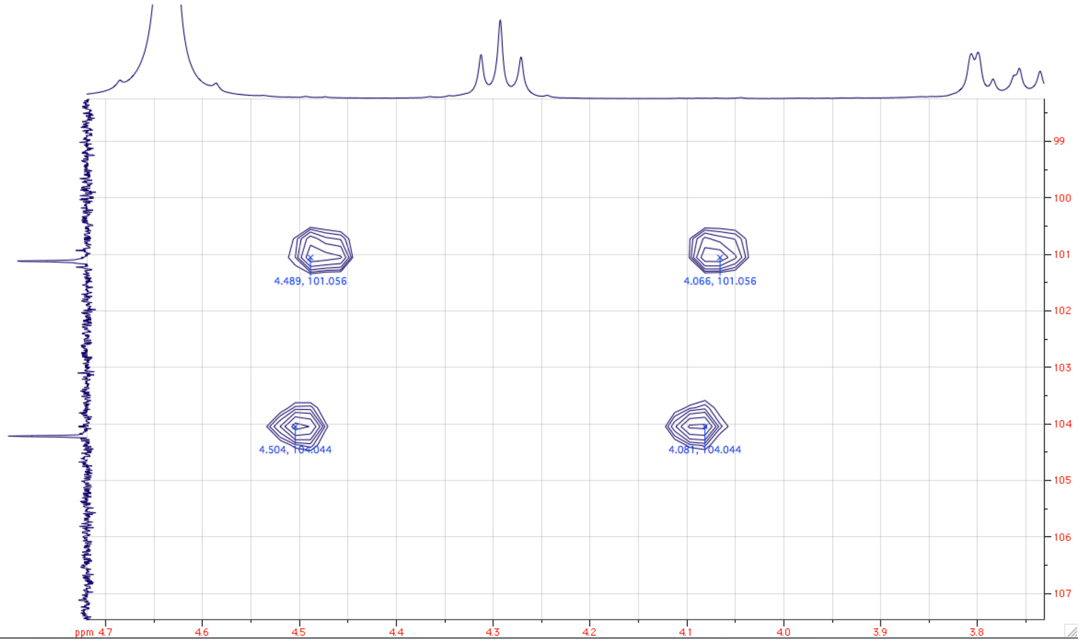

18, ^{1}H NMR, 400 MHz, CDCl$_3$

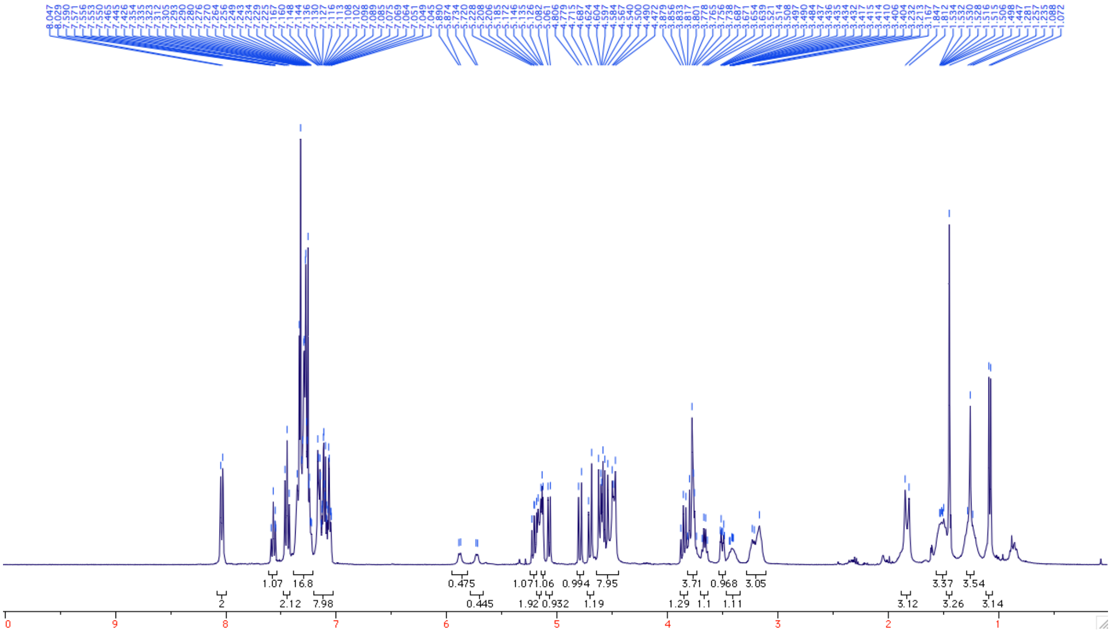

18, COSY, 400 MHz, CDCl$_3$

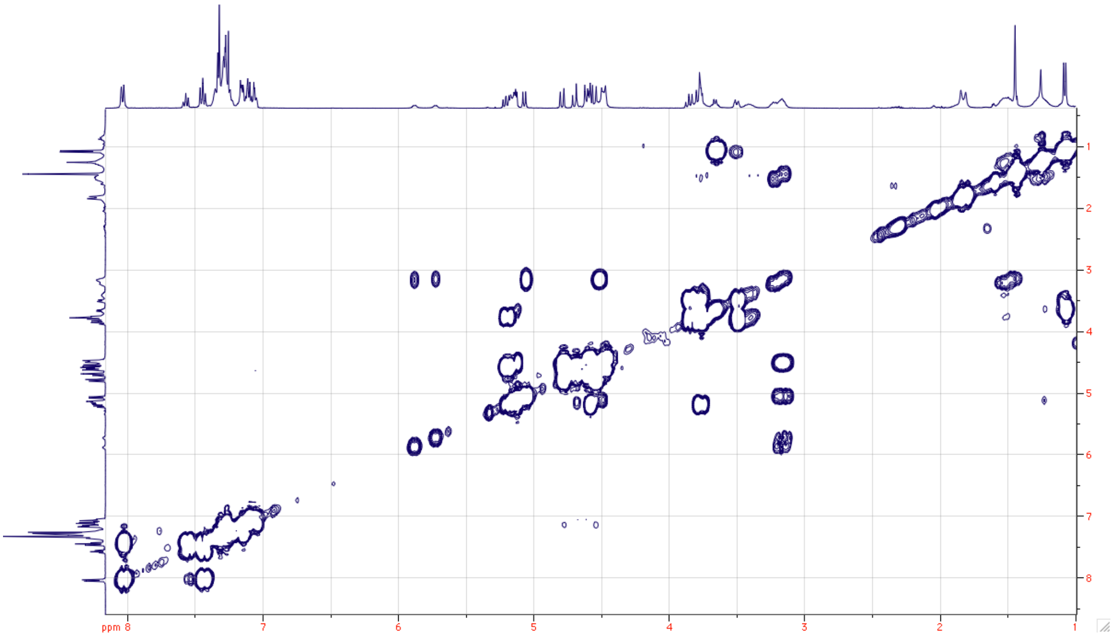

18, ^{13}C NMR, 400 MHz, $CDCl_3$

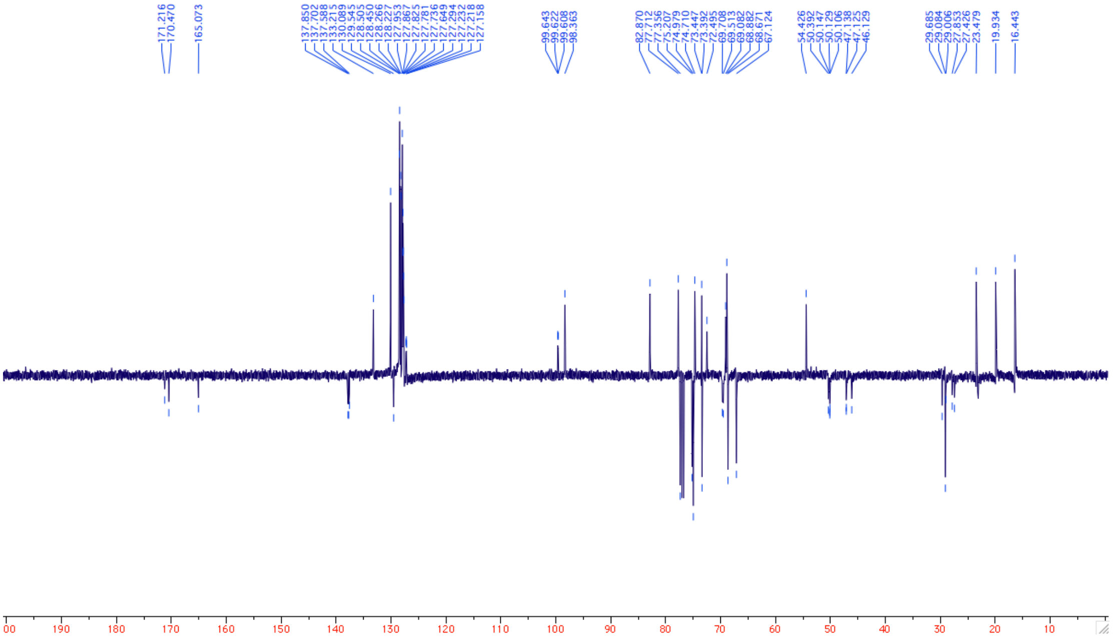

18, HSQC, 400 MHz, $CDCl_3$

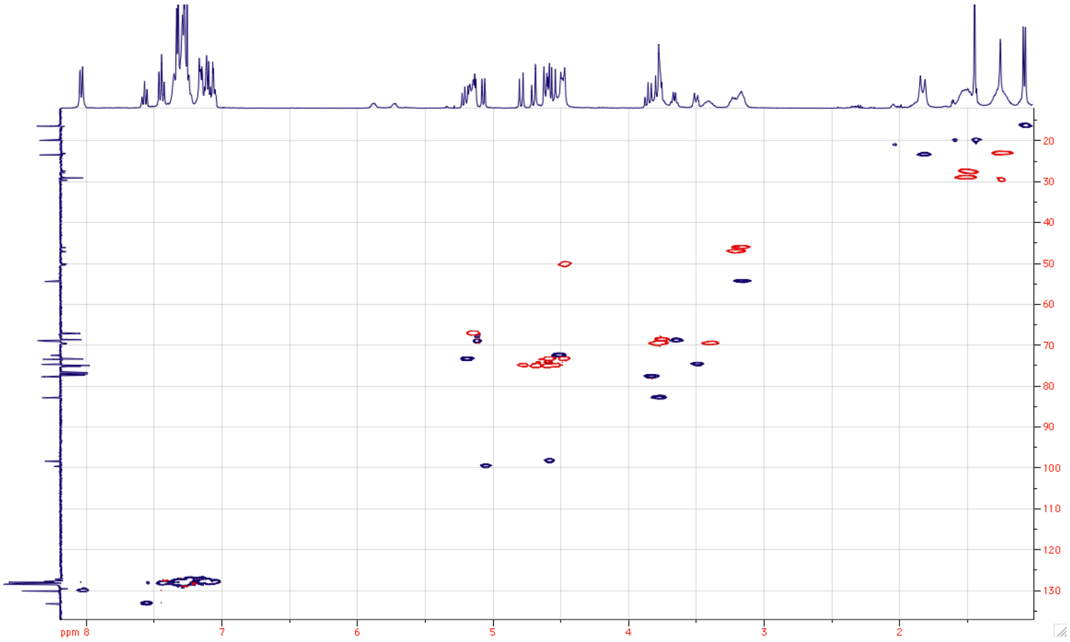

18, decoupled HSQC, 400 MHz, $CDCl_3$

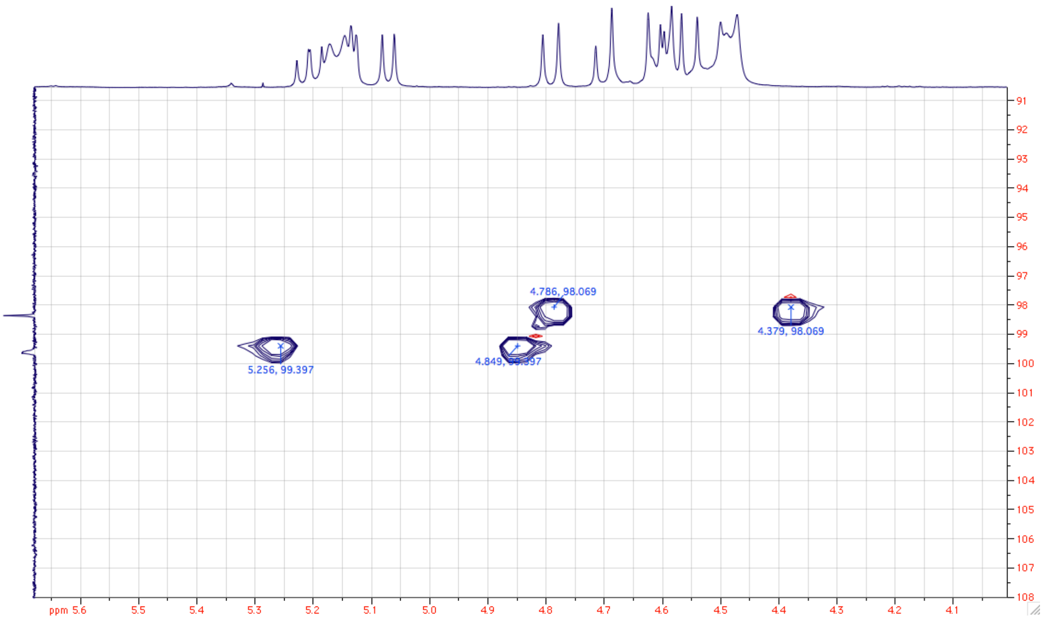

19, ^{1}H NMR, 400 MHz, D_2O

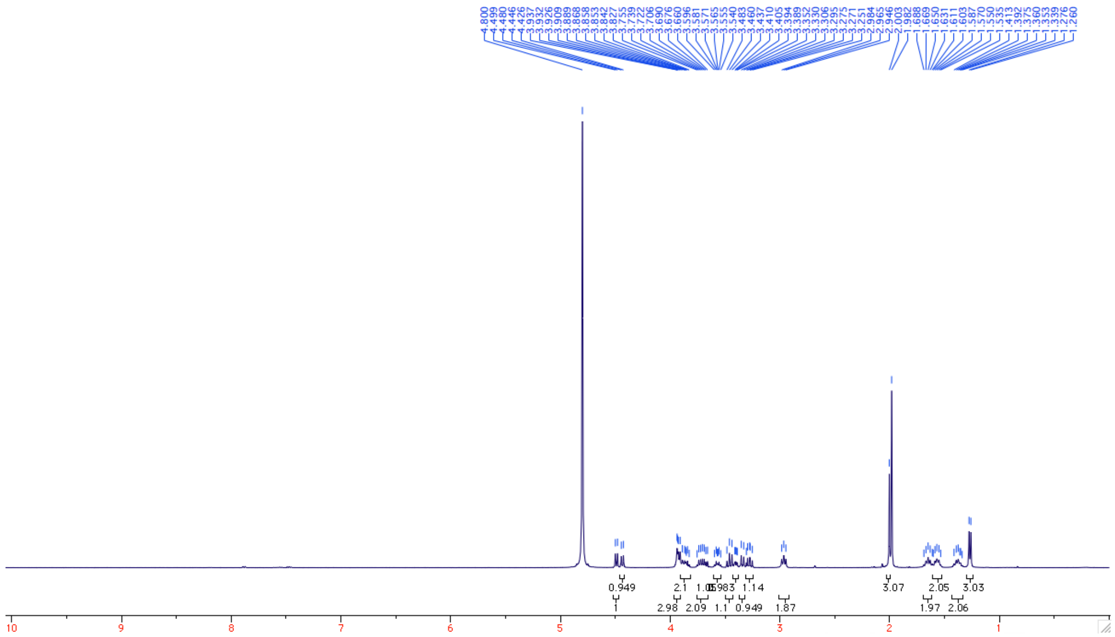

19, COSY NMR, 400 MHz, D_2O

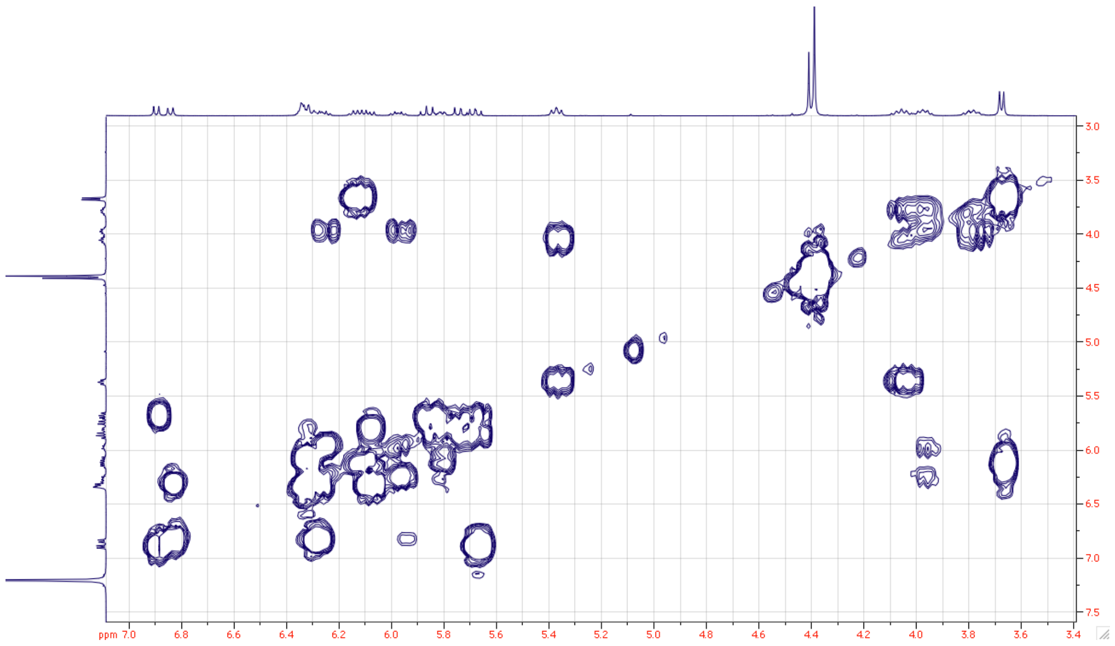

19, ^{13}C NMR, 100 MHz, D_2O

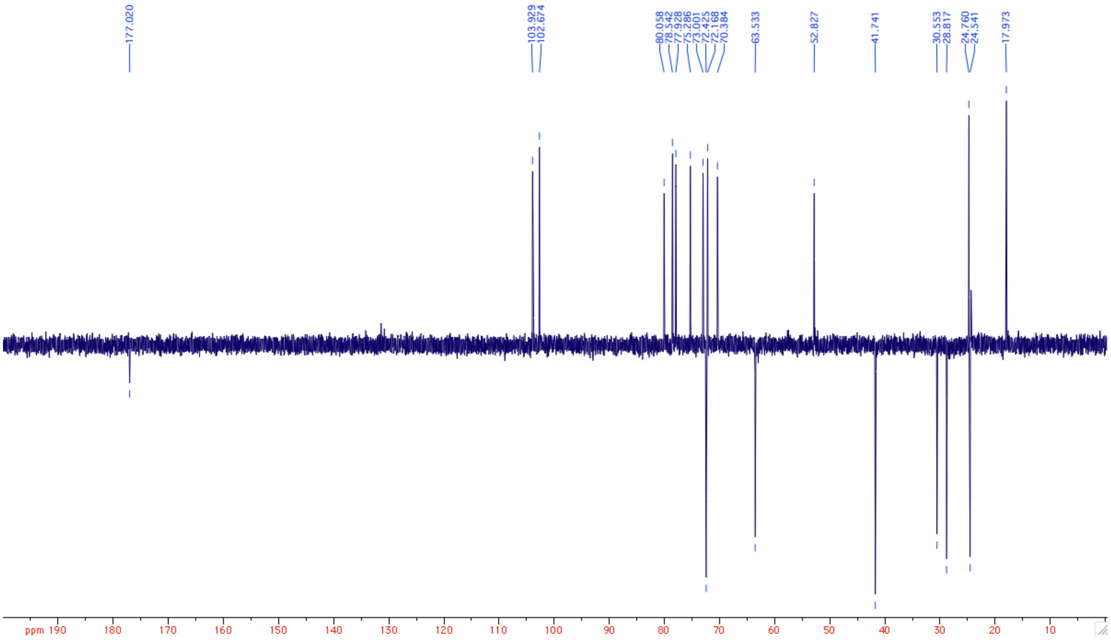

19, HSQC NMR, 400 MHz, D_2O

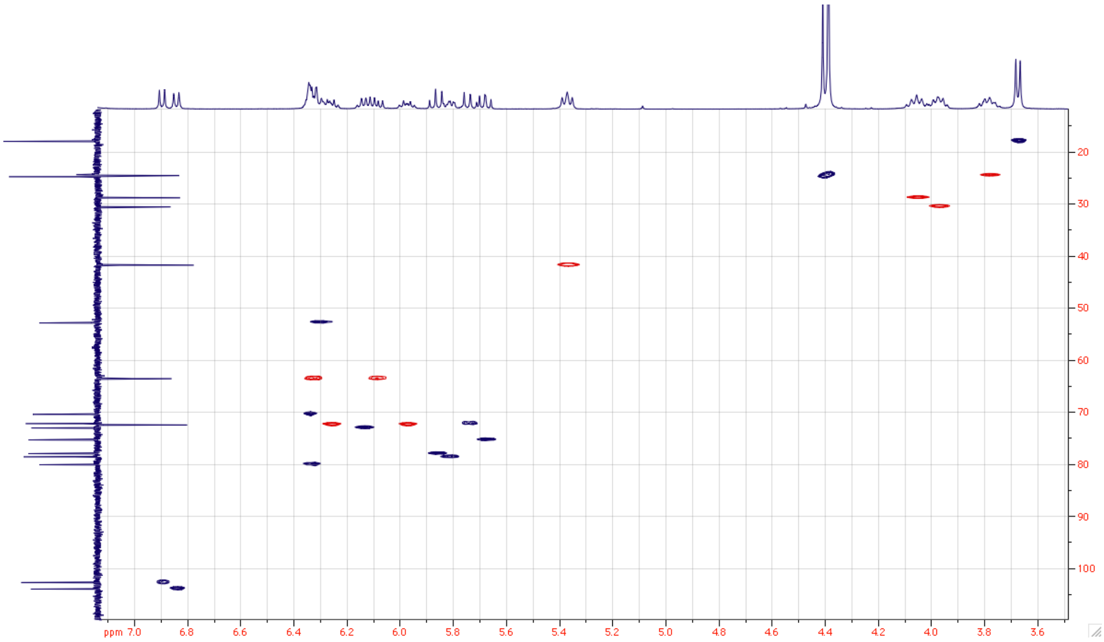

19, coupled HSQC, 400 MHz, D_2O

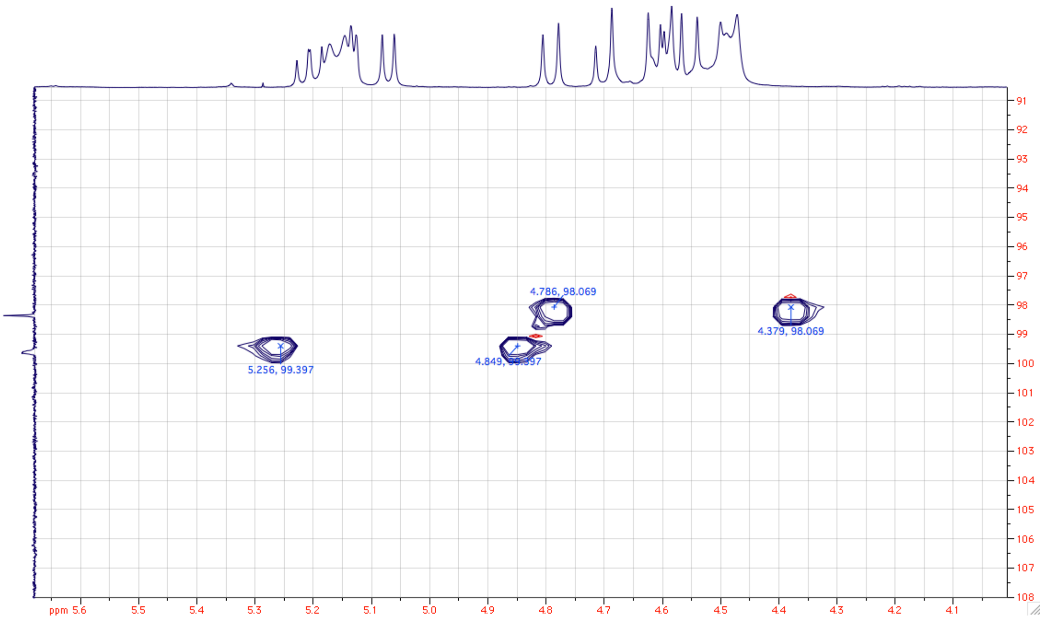

7a, NOESY, 400 MHz, $CDCl_3$

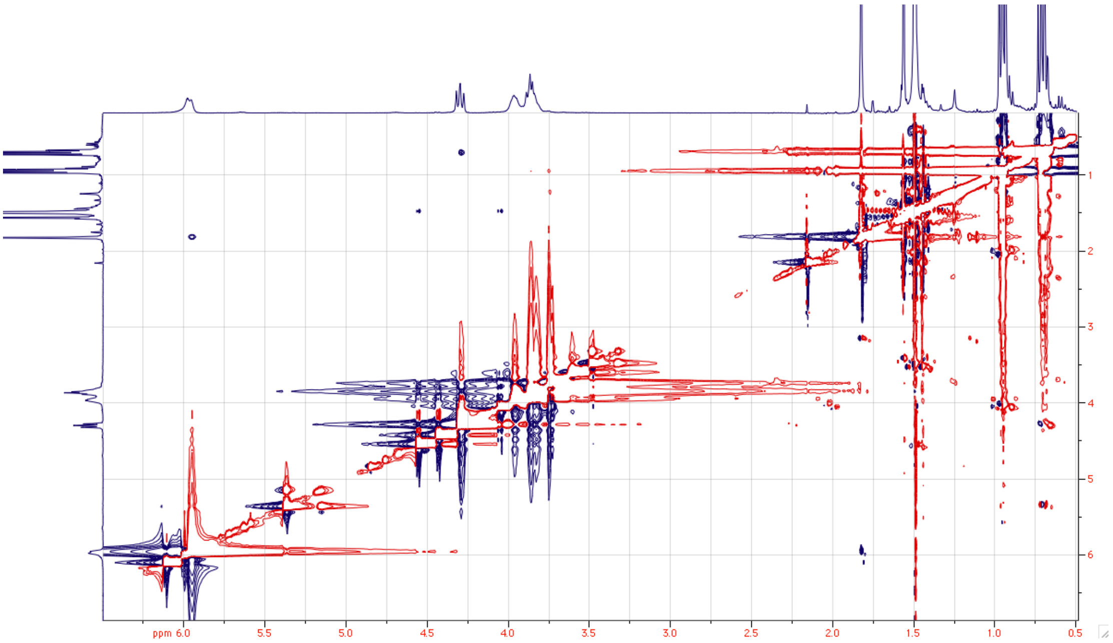

7b, NOESY, 400 MHz, $CDCl_3$

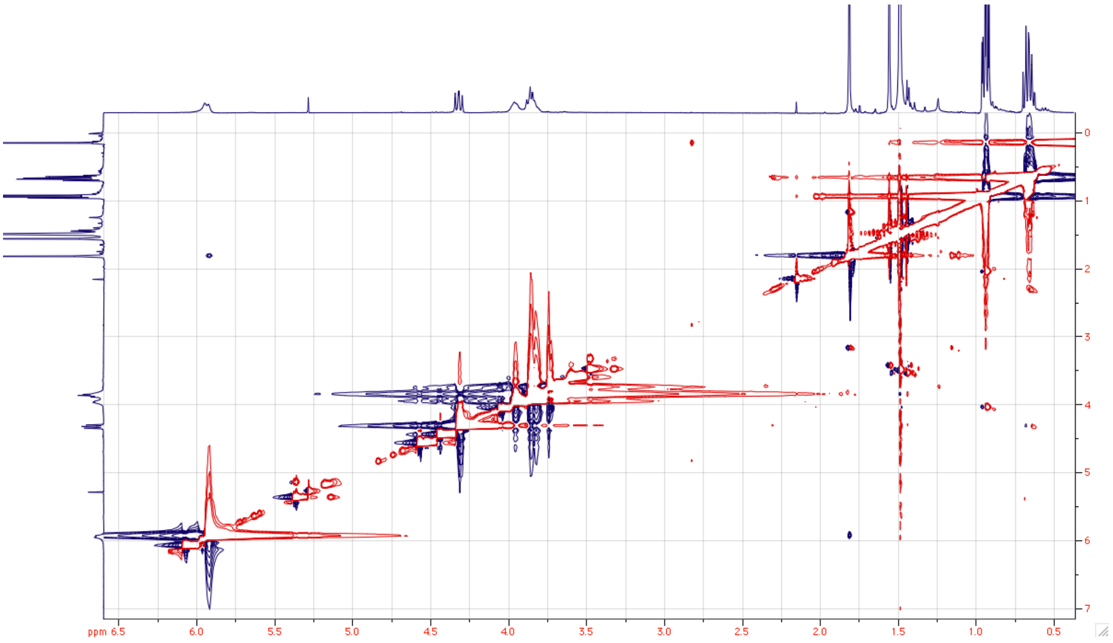

X-ray structure analysis of D-8b

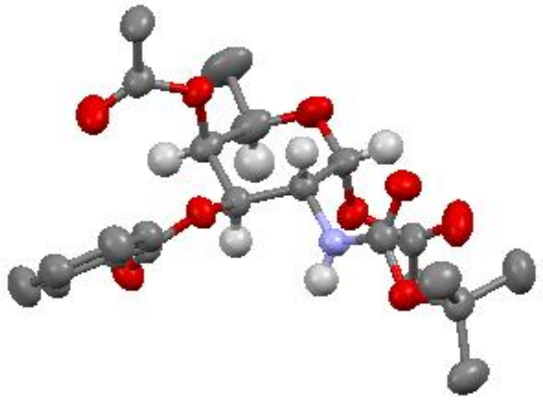

Table 1: Crystal data and structure refinement for D-**8b**.

Identification code	seebdl87	
Name	D-**8b**	
Empirical formula	C44 H58 N2 O18	
Formula weight	902.92	
Temperature	200(2) K	
Wavelength	0.71073 Å	
Radiation	Mo K	
Crystal system	Triclinic	
Space group	P1	
Unit cell dimensions	a = 5.5480(10) Å	α = 90.920(10)°
	b = 9.2950(10) Å	β = 92.610(10)°
	c = 22.922(2) Å	γ = 92.300(10)°
Volume	1179.7(3) $Å^3$	
Z	1	
Density (calculated)	1.271 g/cm^3	
Absorption coefficient	0.099 mm^{-1}	
F(000)	480	
Crystal description	needle	
Crystal colour	colourless	
Crystal size	0.6 x 0.04 x 0.02 mm^3	
No. of reflections (lattice)	17860	
Theta range (lattice)	4.63 to 27.24	
Theta range for data collection	4.62 to 29.27	
Index ranges	-7<=h<=7, -12<=k<=12, -31<=l<=31	
Reflections collected	44527	
Independent reflections	12145 [R(int) = 0.1299]	

Completeness to theta = 29.27°	99.2%
Absorption correction	None
Measurement method	rotation method
Decay	1%
Data collection	STOE X-AREA
Cell refinement	STOE X-AREA
Data reduction	STOE X-RED
Structure solution	SHELXL-97 (Sheldrick, 2008)
Structure refinement	SHELXL-97 (Sheldrick, 2008)
Molecular graphics	ORTEP3
Publication material	WINGX
Refinement method	Full-matrix least-squares on F^2
Weighting scheme	calc
Solution primary	direct
Hydrogen treatment	constr
Data / restraints / parameters	12145 / 3 / 577
Goodness-of-fit on F^2	0.866
Final R indices [I>2sigma(I)]	R1 = 0.0591, wR2 = 0.1223
R indices (all data)	R1 = 0.1474, wR2 = 0.1485
Absolute structure parameter	-0.6(10)
Largest diff. peak and hole	0.178 and -0.208 e.Å^{-3}

Table 2: Atomic coordinates (x 10^4) and equivalent isotropic displacement parameters (Å^2 x 10^3) for D-**8b**. U(eq) is defined as one third of the trace of the orthogonalized U^{ij} tensor.

	x	y	z	U(eq)
C(1)	11528(7)	-98(4)	13578(2)	40(1)
C(2)	9541(7)	-264(4)	14005(2)	42(1)
C(3)	9582(8)	1063(4)	14423(2)	47(1)
O(1)	9512(6)	2329(3)	14132(1)	60(1)
C(4)	11455(10)	2553(4)	13748(2)	63(1)
C(5)	11468(7)	1371(4)	13288(2)	47(1)
C(6)	12868(7)	-1668(4)	12843(2)	42(1)
C(24)	5656(6)	3588(4)	8596(2)	37(1)
C(7)	12080(7)	-2835(4)	12413(2)	40(1)
C(12)	9851(7)	-3534(4)	12438(2)	48(1)
C(40)	7065(7)	2501(4)	7246(2)	47(1)
C(11)	9247(8)	-4642(5)	12031(2)	58(1)
C(10)	10826(8)	-5047(5)	11622(2)	55(1)
C(27)	7724(6)	2058(4)	9327(2)	40(1)
C(26)	7344(8)	794(4)	8900(2)	46(1)
O(12)	11926(4)	4595(3)	9529(1)	53(1)
O(4)	5796(5)	-1885(3)	14507(1)	57(1)
C(28)	10071(6)	5146(4)	9634(2)	40(1)
O(18)	8031(5)	2645(4)	10494(1)	63(1)
C(23)	7859(6)	3450(4)	9007(2)	39(1)
C(29)	9944(7)	6415(4)	10037(2)	40(1)
O(5)	8644(4)	-3369(3)	14879(1)	49(1)
C(43)	6067(7)	2385(4)	10269(2)	42(1)
C(41)	9292(8)	2299(5)	6936(2)	61(1)
O(9)	11305(6)	962(4)	12118(1)	68(1)
N(2)	5837(5)	4916(3)	8263(1)	40(1)
C(42)	6977(10)	-637(4)	9179(2)	63(1)
C(44)	3756(7)	2448(5)	10576(2)	48(1)
C(30)	8032(8)	6634(4)	10368(2)	54(1)
C(35)	3836(7)	5518(4)	8058(2)	40(1)
C(21)	9451(8)	1229(4)	12342(2)	46(1)
C(13)	7876(7)	-2264(4)	14564(2)	46(1)
C(36)	2626(7)	7591(4)	7482(2)	45(1)

C(38)	1123(8)	8283(4)	7941(2)	54(1)
C(22)	7079(7)	1339(5)	12025(2)	57(1)
C(14)	6958(7)	-4310(4)	15190(2)	47(1)
O(3)	14925(5)	-1173(3)	12916(1)	53(1)
C(25)	5387(7)	2271(4)	8193(2)	42(1)
O(17)	5660(4)	2038(2)	9695(1)	38(1)
O(2)	11017(4)	-1237(3)	13149(1)	42(1)
N(1)	9793(6)	-1577(3)	14331(1)	45(1)
O(11)	7881(4)	4677(2)	9414(1)	37(1)
O(6)	11743(5)	1058(3)	14794(1)	48(1)
O(8)	9290(4)	1443(3)	12918(1)	44(1)
O(10)	5297(5)	980(3)	8505(1)	46(1)
O(15)	7433(4)	2234(3)	7822(1)	41(1)
O(14)	4460(4)	6733(2)	7760(1)	45(1)
O(13)	1775(4)	5086(3)	8122(1)	51(1)
O(16)	5187(5)	2873(4)	7041(1)	69(1)
C(39)	4158(8)	8765(5)	7191(2)	61(1)
C(15)	8634(8)	-5344(5)	15488(2)	69(1)
C(34)	11929(8)	7355(5)	10082(2)	64(1)
C(31)	8072(9)	7815(5)	10758(2)	64(1)
C(32)	10061(9)	8760(5)	10788(2)	70(1)
C(8)	13645(8)	-3224(5)	11987(2)	56(1)
C(9)	13002(9)	-4299(5)	11594(2)	65(1)
C(16)	5196(8)	-5071(5)	14764(2)	67(1)
C(37)	1091(9)	6730(5)	7027(2)	65(1)
C(17)	5741(10)	-3420(6)	15648(2)	76(1)
C(33)	11938(9)	8548(5)	10449(3)	82(2)
C(18)	11572(8)	773(5)	15372(2)	50(1)
C(19)	13915(8)	907(5)	15683(2)	63(1)
C(20)	11173(12)	4040(5)	13480(3)	90(2)
O(7)	9668(6)	410(5)	15575(1)	85(1)

Table 3: Bond lengths [Å] and angles [°] for D-**8b**.

C(1)-O(2)	1.443(4)
C(1)-C(2)	1.513(5)
C(1)-C(5)	1.529(5)
C(1)-H(1)	0.9800
C(2)-N(1)	1.449(5)
C(2)-C(3)	1.549(5)
C(2)-H(2)	0.9800
C(3)-O(1)	1.363(5)
C(3)-O(6)	1.438(4)
C(3)-H(3)	0.9800
O(1)-C(4)	1.433(6)
C(4)-C(5)	1.512(6)
C(4)-C(20)	1.533(6)
C(4)-H(4)	0.9800
C(5)-O(8)	1.448(4)
C(5)-H(5)	0.9800
C(6)-O(3)	1.217(4)
C(6)-O(2)	1.341(4)
C(6)-C(7)	1.493(5)
C(24)-N(2)	1.464(5)
C(24)-C(25)	1.519(5)
C(24)-C(23)	1.520(5)
C(24)-H(24)	0.9800
C(7)-C(12)	1.377(5)
C(7)-C(8)	1.388(5)
C(12)-C(11)	1.399(6)
C(12)-H(12)	0.9300
C(40)-O(16)	1.190(4)
C(40)-O(15)	1.355(4)
C(40)-C(41)	1.470(6)
C(11)-C(10)	1.370(6)
C(11)-H(11)	0.9300
C(10)-C(9)	1.373(6)
C(10)-H(10)	0.9300
C(27)-O(17)	1.452(4)
C(27)-C(23)	1.500(5)

C(27)-C(26)	1.520(5)
C(27)-H(27)	0.9800
C(26)-O(10)	1.437(4)
C(26)-C(42)	1.496(6)
C(26)-H(26)	0.9800
O(12)-C(28)	1.200(4)
O(4)-C(13)	1.222(5)
C(28)-O(11)	1.347(4)
C(28)-C(29)	1.493(5)
O(18)-C(43)	1.198(4)
C(23)-O(11)	1.461(4)
C(23)-H(23)	0.9800
C(29)-C(30)	1.352(6)
C(29)-C(34)	1.377(5)
O(5)-C(13)	1.337(5)
O(5)-C(14)	1.471(4)
C(43)-O(17)	1.357(4)
C(43)-C(44)	1.492(5)
C(41)-H(41A)	0.9600
C(41)-H(41B)	0.9600
C(41)-H(41C)	0.9600
O(9)-C(21)	1.203(5)
N(2)-C(35)	1.333(4)
N(2)-H(2A)	0.8600
C(42)-H(42A)	0.9600
C(42)-H(42B)	0.9600
C(42)-H(42C)	0.9600
C(44)-H(44A)	0.9600
C(44)-H(44B)	0.9600
C(44)-H(44C)	0.9600
C(30)-C(31)	1.403(6)
C(30)-H(30)	0.9300
C(35)-O(13)	1.213(4)
C(35)-O(14)	1.369(4)
C(21)-O(8)	1.341(5)
C(21)-C(22)	1.482(5)
C(13)-N(1)	1.354(5)
C(36)-O(14)	1.450(4)

C(36)-C(37)	1.514(5)
C(36)-C(38)	1.522(6)
C(36)-C(39)	1.535(6)
C(38)-H(38A)	0.9600
C(38)-H(38B)	0.9600
C(38)-H(38C)	0.9600
C(22)-H(22A)	0.9600
C(22)-H(22B)	0.9600
C(22)-H(22C)	0.9600
C(14)-C(16)	1.497(5)
C(14)-C(15)	1.513(6)
C(14)-C(17)	1.525(6)
C(25)-O(10)	1.407(4)
C(25)-O(15)	1.450(4)
C(25)-H(25)	0.9800
N(1)-H(1A)	0.8600
O(6)-C(18)	1.363(5)
C(39)-H(39A)	0.9600
C(39)-H(39B)	0.9600
C(39)-H(39C)	0.9600
C(15)-H(15A)	0.9600
C(15)-H(15B)	0.9600
C(15)-H(15C)	0.9600
C(34)-C(33)	1.380(7)
C(34)-H(34)	0.9300
C(31)-C(32)	1.381(7)
C(31)-H(31)	0.9300
C(32)-C(33)	1.347(7)
C(32)-H(32)	0.9300
C(8)-C(9)	1.360(6)
C(8)-H(8)	0.9300
C(9)-H(9)	0.9300
C(16)-H(16A)	0.9600
C(16)-H(16B)	0.9600
C(16)-H(16C)	0.9600
C(37)-H(37A)	0.9600
C(37)-H(37B)	0.9600
C(37)-H(37C)	0.9600

C(17)-H(17A)	0.9600
C(17)-H(17B)	0.9600
C(17)-H(17C)	0.9600
C(33)-H(33)	0.9300
C(18)-O(7)	1.211(5)
C(18)-C(19)	1.453(6)
C(19)-H(19A)	0.9600
C(19)-H(19B)	0.9600
C(19)-H(19C)	0.9600
C(20)-H(20A)	0.9600
C(20)-H(20B)	0.9600
C(20)-H(20C)	0.9600
O(2)-C(1)-C(2)	104.8(3)
O(2)-C(1)-C(5)	110.4(3)
C(2)-C(1)-C(5)	110.8(3)
O(2)-C(1)-H(1)	110.3
C(2)-C(1)-H(1)	110.3
C(5)-C(1)-H(1)	110.3
N(1)-C(2)-C(1)	110.4(3)
N(1)-C(2)-C(3)	110.8(3)
C(1)-C(2)-C(3)	110.2(3)
N(1)-C(2)-H(2)	108.5
C(1)-C(2)-H(2)	108.5
C(3)-C(2)-H(2)	108.5
O(1)-C(3)-O(6)	109.5(3)
O(1)-C(3)-C(2)	112.5(3)
O(6)-C(3)-C(2)	108.5(3)
O(1)-C(3)-H(3)	108.7
O(6)-C(3)-H(3)	108.7
C(2)-C(3)-H(3)	108.7
C(3)-O(1)-C(4)	113.8(3)
O(1)-C(4)-C(5)	111.6(4)
O(1)-C(4)-C(20)	107.2(4)
C(5)-C(4)-C(20)	112.1(4)
O(1)-C(4)-H(4)	108.7
C(5)-C(4)-H(4)	108.7
C(20)-C(4)-H(4)	108.7

O(8)-C(5)-C(4)	108.3(3)
O(8)-C(5)-C(1)	109.6(3)
C(4)-C(5)-C(1)	110.0(3)
O(8)-C(5)-H(5)	109.7
C(4)-C(5)-H(5)	109.7
C(1)-C(5)-H(5)	109.7
O(3)-C(6)-O(2)	123.7(3)
O(3)-C(6)-C(7)	124.9(4)
O(2)-C(6)-C(7)	111.4(3)
N(2)-C(24)-C(25)	111.3(3)
N(2)-C(24)-C(23)	110.9(3)
C(25)-C(24)-C(23)	109.3(3)
N(2)-C(24)-H(24)	108.4
C(25)-C(24)-H(24)	108.4
C(23)-C(24)-H(24)	108.4
C(12)-C(7)-C(8)	120.1(4)
C(12)-C(7)-C(6)	121.0(3)
C(8)-C(7)-C(6)	118.9(3)
C(7)-C(12)-C(11)	118.1(4)
C(7)-C(12)-H(12)	120.9
C(11)-C(12)-H(12)	120.9
O(16)-C(40)-O(15)	122.8(4)
O(16)-C(40)-C(41)	126.8(4)
O(15)-C(40)-C(41)	110.4(3)
C(10)-C(11)-C(12)	121.5(4)
C(10)-C(11)-H(11)	119.3
C(12)-C(11)-H(11)	119.3
C(11)-C(10)-C(9)	119.1(4)
C(11)-C(10)-H(10)	120.5
C(9)-C(10)-H(10)	120.5
O(17)-C(27)-C(23)	109.5(3)
O(17)-C(27)-C(26)	107.0(3)
C(23)-C(27)-C(26)	110.5(3)
O(17)-C(27)-H(27)	109.9
C(23)-C(27)-H(27)	109.9
C(26)-C(27)-H(27)	109.9
O(10)-C(26)-C(42)	107.1(3)
O(10)-C(26)-C(27)	111.4(3)

C(42)-C(26)-C(27)	114.6(3)
O(10)-C(26)-H(26)	107.8
C(42)-C(26)-H(26)	107.8
C(27)-C(26)-H(26)	107.8
O(12)-C(28)-O(11)	124.1(3)
O(12)-C(28)-C(29)	123.2(3)
O(11)-C(28)-C(29)	112.6(3)
O(11)-C(23)-C(27)	110.8(3)
O(11)-C(23)-C(24)	106.4(3)
C(27)-C(23)-C(24)	110.9(3)
O(11)-C(23)-H(23)	109.6
C(27)-C(23)-H(23)	109.6
C(24)-C(23)-H(23)	109.6
C(30)-C(29)-C(34)	119.9(4)
C(30)-C(29)-C(28)	122.9(3)
C(34)-C(29)-C(28)	117.1(4)
C(13)-O(5)-C(14)	121.6(3)
O(18)-C(43)-O(17)	124.0(4)
O(18)-C(43)-C(44)	124.7(4)
O(17)-C(43)-C(44)	111.3(3)
C(40)-C(41)-H(41A)	109.5
C(40)-C(41)-H(41B)	109.5
H(41A)-C(41)-H(41B)	109.5
C(40)-C(41)-H(41C)	109.5
H(41A)-C(41)-H(41C)	109.5
H(41B)-C(41)-H(41C)	109.5
C(35)-N(2)-C(24)	119.8(3)
C(35)-N(2)-H(2A)	120.1
C(24)-N(2)-H(2A)	120.1
C(26)-C(42)-H(42A)	109.5
C(26)-C(42)-H(42B)	109.5
H(42A)-C(42)-H(42B)	109.5
C(26)-C(42)-H(42C)	109.5
H(42A)-C(42)-H(42C)	109.5
H(42B)-C(42)-H(42C)	109.5
C(43)-C(44)-H(44A)	109.5
C(43)-C(44)-H(44B)	109.5
H(44A)-C(44)-H(44B)	109.5

C(43)-C(44)-H(44C)	109.5
H(44A)-C(44)-H(44C)	109.5
H(44B)-C(44)-H(44C)	109.5
C(29)-C(30)-C(31)	120.1(4)
C(29)-C(30)-H(30)	120.0
C(31)-C(30)-H(30)	120.0
O(13)-C(35)-N(2)	126.5(3)
O(13)-C(35)-O(14)	124.4(3)
N(2)-C(35)-O(14)	109.1(3)
O(9)-C(21)-O(8)	123.3(4)
O(9)-C(21)-C(22)	125.0(4)
O(8)-C(21)-C(22)	111.7(4)
O(4)-C(13)-O(5)	126.3(4)
O(4)-C(13)-N(1)	124.4(4)
O(5)-C(13)-N(1)	109.2(3)
O(14)-C(36)-C(37)	112.0(3)
O(14)-C(36)-C(38)	110.2(3)
C(37)-C(36)-C(38)	112.5(3)
O(14)-C(36)-C(39)	102.0(3)
C(37)-C(36)-C(39)	110.5(4)
C(38)-C(36)-C(39)	109.2(3)
C(36)-C(38)-H(38A)	109.5
C(36)-C(38)-H(38B)	109.5
H(38A)-C(38)-H(38B)	109.5
C(36)-C(38)-H(38C)	109.5
H(38A)-C(38)-H(38C)	109.5
H(38B)-C(38)-H(38C)	109.5
C(21)-C(22)-H(22A)	109.5
C(21)-C(22)-H(22B)	109.5
H(22A)-C(22)-H(22B)	109.5
C(21)-C(22)-H(22C)	109.5
H(22A)-C(22)-H(22C)	109.5
H(22B)-C(22)-H(22C)	109.5
O(5)-C(14)-C(16)	110.2(3)
O(5)-C(14)-C(15)	102.3(3)
C(16)-C(14)-C(15)	111.8(4)
O(5)-C(14)-C(17)	109.2(3)
C(16)-C(14)-C(17)	113.1(4)

C(15)-C(14)-C(17)	109.7(4)
O(10)-C(25)-O(15)	107.8(3)
O(10)-C(25)-C(24)	112.1(3)
O(15)-C(25)-C(24)	109.6(3)
O(10)-C(25)-H(25)	109.1
O(15)-C(25)-H(25)	109.1
C(24)-C(25)-H(25)	109.1
C(43)-O(17)-C(27)	117.7(3)
C(6)-O(2)-C(1)	117.1(3)
C(13)-N(1)-C(2)	121.8(3)
C(13)-N(1)-H(1A)	119.1
C(2)-N(1)-H(1A)	119.1
C(28)-O(11)-C(23)	115.9(3)
C(18)-O(6)-C(3)	119.4(3)
C(21)-O(8)-C(5)	118.2(3)
C(25)-O(10)-C(26)	114.5(3)
C(40)-O(15)-C(25)	118.4(3)
C(35)-O(14)-C(36)	120.8(3)
C(36)-C(39)-H(39A)	109.5
C(36)-C(39)-H(39B)	109.5
H(39A)-C(39)-H(39B)	109.5
C(36)-C(39)-H(39C)	109.5
H(39A)-C(39)-H(39C)	109.5
H(39B)-C(39)-H(39C)	109.5
C(14)-C(15)-H(15A)	109.5
C(14)-C(15)-H(15B)	109.5
H(15A)-C(15)-H(15B)	109.5
C(14)-C(15)-H(15C)	109.5
H(15A)-C(15)-H(15C)	109.5
H(15B)-C(15)-H(15C)	109.5
C(29)-C(34)-C(33)	120.2(5)
C(29)-C(34)-H(34)	119.9
C(33)-C(34)-H(34)	119.9
C(32)-C(31)-C(30)	119.1(5)
C(32)-C(31)-H(31)	120.5
C(30)-C(31)-H(31)	120.5
C(33)-C(32)-C(31)	120.4(5)
C(33)-C(32)-H(32)	119.8

C(31)-C(32)-H(32)	119.8
C(9)-C(8)-C(7)	120.5(4)
C(9)-C(8)-H(8)	119.7
C(7)-C(8)-H(8)	119.7
C(8)-C(9)-C(10)	120.6(4)
C(8)-C(9)-H(9)	119.7
C(10)-C(9)-H(9)	119.7
C(14)-C(16)-H(16A)	109.5
C(14)-C(16)-H(16B)	109.5
H(16A)-C(16)-H(16B)	109.5
C(14)-C(16)-H(16C)	109.5
H(16A)-C(16)-H(16C)	109.5
H(16B)-C(16)-H(16C)	109.5
C(36)-C(37)-H(37A)	109.5
C(36)-C(37)-H(37B)	109.5
H(37A)-C(37)-H(37B)	109.5
C(36)-C(37)-H(37C)	109.5
H(37A)-C(37)-H(37C)	109.5
H(37B)-C(37)-H(37C)	109.5
C(14)-C(17)-H(17A)	109.5
C(14)-C(17)-H(17B)	109.5
H(17A)-C(17)-H(17B)	109.5
C(14)-C(17)-H(17C)	109.5
H(17A)-C(17)-H(17C)	109.5
H(17B)-C(17)-H(17C)	109.5
C(32)-C(33)-C(34)	120.3(4)
C(32)-C(33)-H(33)	119.9
C(34)-C(33)-H(33)	119.9
O(7)-C(18)-O(6)	121.7(4)
O(7)-C(18)-C(19)	126.7(4)
O(6)-C(18)-C(19)	111.6(4)
C(18)-C(19)-H(19A)	109.5
C(18)-C(19)-H(19B)	109.5
H(19A)-C(19)-H(19B)	109.5
C(18)-C(19)-H(19C)	109.5
H(19A)-C(19)-H(19C)	109.5
H(19B)-C(19)-H(19C)	109.5
C(4)-C(20)-H(20A)	109.5

C(4)-C(20)-H(20B)	109.5
H(20A)-C(20)-H(20B)	109.5
C(4)-C(20)-H(20C)	109.5
H(20A)-C(20)-H(20C)	109.5
H(20B)-C(20)-H(20C)	109.5

Symmetry transformations used to generate equivalent atoms:

Table 4: Anisotropic displacement parameters (Å^2 x 10^3)for D-**8b**. The anisotropic displacement factor exponent takes the form: $-2p^2[h^2a^{*2}U^{11} + ... + 2 h k a^* b^* U^{12}]$

	U^{11}	U^{22}	U^{33}	U^{23}	U^{13}	U^{12}
C(1)	48(2)	38(2)	35(2)	-7(2)	-2(2)	-3(2)
C(2)	41(2)	42(2)	41(2)	2(2)	0(2)	3(2)
C(3)	55(2)	51(2)	36(2)	-1(2)	-9(2)	10(2)
O(1)	83(2)	44(2)	51(2)	0(1)	-9(2)	14(2)
C(4)	85(3)	46(3)	56(3)	-1(2)	-21(3)	-2(2)
C(5)	49(2)	48(2)	42(2)	5(2)	-12(2)	-6(2)
C(6)	41(2)	50(2)	35(2)	2(2)	0(2)	-1(2)
C(24)	34(2)	41(2)	35(2)	1(2)	1(2)	-2(2)
C(7)	38(2)	43(2)	40(2)	-3(2)	1(2)	1(2)
C(12)	49(2)	46(2)	49(2)	-3(2)	5(2)	7(2)
C(40)	40(2)	59(3)	40(2)	7(2)	-5(2)	0(2)
C(11)	53(3)	56(3)	63(3)	-11(2)	1(2)	-6(2)
C(10)	63(3)	53(2)	49(2)	-11(2)	-3(2)	8(2)
C(27)	37(2)	44(2)	41(2)	-3(2)	8(2)	6(2)
C(26)	60(3)	39(2)	38(2)	-1(2)	0(2)	6(2)
O(12)	28(1)	77(2)	53(2)	-14(1)	1(1)	7(1)
O(4)	39(2)	65(2)	66(2)	11(2)	-1(1)	6(1)
C(28)	32(2)	46(2)	42(2)	5(2)	0(2)	-4(2)
O(18)	40(2)	106(2)	41(2)	-11(2)	-4(1)	-4(2)
C(23)	32(2)	44(2)	42(2)	-4(2)	2(2)	2(2)
C(29)	40(2)	39(2)	40(2)	-4(2)	0(2)	4(2)
O(5)	42(2)	51(2)	53(2)	9(1)	1(1)	-3(1)
C(43)	41(2)	42(2)	41(2)	1(2)	1(2)	1(2)
C(41)	65(3)	82(3)	38(2)	2(2)	8(2)	18(2)
O(9)	50(2)	108(3)	46(2)	-1(2)	-3(2)	4(2)
N(2)	33(2)	40(2)	47(2)	6(1)	1(1)	-2(1)
C(42)	95(4)	38(2)	55(3)	1(2)	2(2)	6(2)
C(44)	45(2)	61(3)	39(2)	-3(2)	9(2)	6(2)
C(30)	54(3)	50(2)	59(3)	-5(2)	5(2)	-4(2)
C(35)	38(2)	42(2)	38(2)	3(2)	-1(2)	-1(2)
C(21)	48(2)	49(2)	40(2)	2(2)	-4(2)	-5(2)
C(13)	45(2)	48(2)	44(2)	-5(2)	1(2)	-4(2)
C(36)	45(2)	42(2)	47(2)	3(2)	-4(2)	6(2)

C(38)	57(3)	43(2)	62(3)	-6(2)	4(2)	9(2)
C(22)	49(2)	75(3)	46(2)	2(2)	-7(2)	-6(2)
C(14)	44(2)	49(2)	47(2)	3(2)	3(2)	-10(2)
O(3)	40(2)	62(2)	55(2)	-11(1)	5(1)	-2(1)
C(25)	41(2)	43(2)	43(2)	1(2)	4(2)	1(2)
O(17)	36(1)	43(1)	35(1)	-3(1)	1(1)	-2(1)
O(2)	37(1)	45(1)	43(2)	-9(1)	1(1)	-4(1)
N(1)	38(2)	48(2)	49(2)	6(2)	4(1)	8(1)
O(11)	32(1)	39(1)	39(1)	-7(1)	2(1)	0(1)
O(6)	51(2)	54(2)	37(2)	1(1)	-4(1)	-3(1)
O(8)	50(2)	46(2)	34(1)	-1(1)	-6(1)	-2(1)
O(10)	59(2)	36(1)	42(2)	-2(1)	3(1)	-5(1)
O(15)	39(1)	53(2)	32(1)	-1(1)	4(1)	7(1)
O(14)	41(1)	40(1)	53(2)	10(1)	4(1)	3(1)
O(13)	35(1)	52(2)	66(2)	18(1)	1(1)	-4(1)
O(16)	50(2)	114(3)	43(2)	12(2)	-5(1)	9(2)
C(39)	60(3)	50(2)	74(3)	25(2)	7(2)	10(2)
C(15)	63(3)	66(3)	77(3)	25(2)	-8(2)	-9(2)
C(34)	38(2)	60(3)	94(4)	-16(2)	2(2)	-2(2)
C(31)	65(3)	70(3)	56(3)	-14(2)	10(2)	4(2)
C(32)	72(3)	63(3)	72(3)	-15(2)	-16(3)	1(2)
C(8)	49(2)	62(3)	57(3)	-13(2)	7(2)	2(2)
C(9)	66(3)	73(3)	55(3)	-22(2)	9(2)	4(2)
C(16)	60(3)	59(3)	79(3)	6(2)	-11(2)	-17(2)
C(37)	71(3)	69(3)	53(3)	-6(2)	-16(2)	-5(2)
C(17)	81(4)	86(4)	61(3)	-6(3)	19(3)	0(3)
C(33)	55(3)	63(3)	123(5)	-45(3)	3(3)	-14(2)
C(18)	55(3)	60(3)	35(2)	-6(2)	0(2)	3(2)
C(19)	62(3)	78(3)	45(2)	6(2)	-11(2)	-8(2)
C(20)	139(5)	42(3)	86(4)	10(3)	-29(4)	-2(3)
O(7)	65(2)	141(3)	48(2)	5(2)	7(2)	-1(2)

Table 5: Hydrogen coordinates (x 10^4) and isotropic displacement parameters ($Å^2$x 10^3) for seebdl87.

	x	y	z	U(eq)
H(1)	13106	-219	13777	49
H(2)	7984	-323	13784	50
H(3)	8179	982	14667	57
H(4)	12985	2560	13980	76
H(5)	12886	1511	13052	56
H(24)	4224	3620	8830	44
H(12)	8778	-3276	12718	57
H(11)	7739	-5114	12037	69
H(10)	10431	-5817	11367	66
H(27)	9211	1951	9567	48
H(26)	8780	752	8668	55
H(23)	9324	3498	8783	47
H(41A)	10540	2004	7207	92
H(41B)	9792	3190	6763	92
H(41C)	8999	1573	6636	92
H(2A)	7230	5306	8200	48
H(42A)	6758	-1375	8881	94
H(42B)	5573	-626	9408	94
H(42C)	8367	-828	9426	94
H(44A)	2426	2205	10305	72
H(44B)	3590	3403	10730	72
H(44C)	3766	1775	10889	72
H(30)	6689	6001	10337	65
H(38A)	2171	8814	8219	81
H(38B)	227	7547	8138	81
H(38C)	24	8924	7755	81
H(22A)	5886	1583	12296	85
H(22B)	6622	432	11837	85
H(22C)	7194	2072	11736	85
H(25)	3901	2329	7948	51
H(1A)	11197	-1929	14377	53
H(39A)	5127	9297	7485	91

H(39B)	3115	9406	6986	91
H(39C)	5189	8326	6920	91
H(15A)	9731	-4820	15757	104
H(15B)	9531	-5824	15199	104
H(15C)	7702	-6044	15695	104
H(34)	13267	7186	9865	77
H(31)	6776	7960	10993	76
H(32)	10104	9548	11044	83
H(8)	15145	-2746	11971	67
H(9)	14046	-4529	11303	78
H(16A)	6058	-5608	14486	100
H(16B)	4244	-4376	14564	100
H(16C)	4156	-5716	14970	100
H(37A)	142	6003	7215	98
H(37B)	2117	6282	6758	98
H(37C)	39	7361	6819	98
H(17A)	6951	-2949	15905	113
H(17B)	4718	-4040	15869	113
H(17C)	4787	-2710	15458	113
H(33)	13246	9207	10462	98
H(19A)	15114	1229	15420	94
H(19B)	14329	-12	15835	94
H(19C)	13850	1591	15999	94
H(20A)	11172	4758	13785	135
H(20B)	9679	4046	13252	135
H(20C)	12493	4247	13233	135
